# COVID-19 and drivers of excess death rate in Peru: A longitudinal ecological study

**DOI:** 10.1016/j.heliyon.2022.e11948

**Published:** 2022-11-30

**Authors:** Kim N. Cajachagua-Torres, Hugo G. Quezada-Pinedo, Carlos A. Huayanay-Espinoza, Jordan A. Obeso-Manrique, Víctor A. Peña-Rodríguez, Elisa Vidal, Luis Huicho

**Affiliations:** a The Generation R Study Group, Erasmus MC, University Medical Centre Rotterdam, 3000, CA, Rotterdam, the Netherlands; b Centro de Investigación en Salud Materna e Infantil and Centro de Investigación para el Desarrollo Integral y Sostenible, Universidad Peruana Cayetano Heredia, 150135, Lima, Peru; c Facultad de Ciencias Físicas, Universidad Nacional Mayor de San Marcos, 15081, Lima, Peru; d Centro de Investigaciones Tecnológicas, Biomédicas y Medioambientales, 15081, Lima, Peru; e Facultad de Medicina “Alberto Hurtado”, Universidad Peruana Cayetano Heredia, 150135, Lima, Peru

**Keywords:** COVID-19, Pandemic, Excess death, Mortality, Inequity, Peru

## Abstract

**Background:**

Peru has experienced unprecedented mortality and economic toll due to the COVID-19 (Coronavirus disease 2019) pandemic in 2020. We aimed to assess the association between socioeconomic factors and excess death rate, and to explore the relative contribution of these factors to the differences in excess death rate during January–December 2020.

**Methods:**

Different national secondary data sources were used to describe excess death rates and different determinants, from distal to proximal. A confounding-adjusted multilevel mixed-effects linear regression was used to assess the association between these variables and excess death rates. Their relative contributions to the differences in excess death rate between the periods with the highest and lowest excess death rates were analyzed through regression-based Oaxaca-Blinder decomposition methods.

**Findings:**

The excess death rate showed an increasing trend in all regions, with different slopes. The confounding-adjusted multilevel analysis showed that higher healthcare access was associated with lower excess death rates (difference (95%CI) -0.004 (-0.005, -0.002)), whereas COVID-19 incidence was associated with higher excess death rates (difference (95%CI) 0.052 (0.042, 0.063)). The decomposition analysis showed COVID-19 incidence (41.9%), per capita income (19.4%) and unemployment rate (14.6%) as the main risk factors, while the main protective factors included per capita health expenditure (44.7%), healthcare access (33.2%) and health insurance (12.1%).

**Interpretation:**

Our study suggests that the excess death rate during the COVID-19 pandemic in Peru may have been influenced by other factors besides COVID-19 incidence, from distal to proximal drivers, including socioeconomic determinants, factors outside and within the health sector, and susceptibility factors. Further studies at individual level are needed to corroborate our findings.

## 1 Introduction

COVID-19 (Coronavirus disease 2019) is caused by SARS-CoV-2, a beta-coronavirus that belongs to the *Coronaviridae* family (Sohrabi et al., 2020). SARS-CoV-2 causes a severe acute respiratory infection (Huang et al., 2020), and was initially identified in Wuhan city, Hubei province, China (Sohrabi et al., 2020). The most common symptoms include fever, cough, myalgia, anosmia, ageusia and dyspnoea (Huang et al., 2020). The World Health Organization (WHO) declared the COVID-19 as a Public Health Emergency of International Concern on 30^th^ January 2020 (de Souza et al., 2020; Sohrabi et al., 2020). By 31 December 2020, 82, 839, 987 cases and 1,901,444 deaths due COVID-19 had been reported worldwide (World Health Organization, 2021). In the same period South America reported 15, 575, 630 cases and 583,400 deaths due COVID-19, of which Peru reported 1,010,496 cases and 93,066 deaths, accounting for 6.5% of cases and 16.0% of deaths in the region (World Health Organization, 2021). However, when considering the excess death rate as an indicator, Peru has been one of the worst hit countries worldwide (Financial Times, 2021). The excess death rate has been proposed as a better tool than COVID-19 deaths to assess the direct and indirect effects of COVID-19 on mortality (Feyman et al., 2022; Henry et al., 2022; Islam et al., 2021; Iuliano et al., 2021; Kontis et al., 2020; Kontopantelis et al., 2021; Lewnard et al., 2021; Moghadas and Galvani, 2021; Sempé et al., 2021).

Peru confirmed the first COVID-19 case on 6^th^ March 2020, declared National Health Emergency on 15^th^ March 2020 and closed its borders the following day (Munayco et al., 2020). Even though many strategies were implemented by the government in early stages of the pandemic, the number of cases and deaths increased rapidly (Munayco et al., 2020). Peru has enjoyed continued economic growth over the last two decades, but inequities have remained, and have been blamed as one of key factors contributing to mortality during the pandemic (Archiniegas, 2020; Goutte et al., 2020). The pandemic found a country with persistent social and economic inequities (Santos et al., 2021). Thus, country efforts to systematically disentangle the factors associated with the excess death rates during the COVID-19 pandemic are largely warranted (Kapitsinis, 2021).

Therefore, in this study we aimed to assess, at the regional level and during the period January–December 2020, the association between different factors and excess death rate, and to explore their relative contribution to the differences in excess death between the period with the lowest excess death rates and the period with the highest excess death rates.

## 2 Methods

### 2.1 Study design and setting

A longitudinal ecological study was performed, which combined the investigation of geographical information about the twenty-five political and administrative regions from Peru and individual level information collected through the Peruvian national death register, SINADEF (*Sistema Informático Nacional de Defunciones* in Spanish). It covered the period from January 2020 to December 2020. Peru is an upper-middle income country with wide differences in economic, social, geographic and cultural characteristics (The World Bank, 2021). It is geographically divided in three natural regions (namely Coast, Highlands and Amazon), and it has twenty-five political and administrative regions. The central government has decentralized important functions to the twenty-five regional governments, although this is a largely unfinished process with mixed results (Loayza et al., 2014). Regional governments hold political, administrative and economic responsibilities for each region, with a high degree of autonomy (Loayza et al., 2014). However, the central government retains the final decision on budget allocation to regions (Loayza et al., 2014). A summary of the principal COVID-19 related measures implemented by the Peruvian government during 2020 is shown in the Supplementary material 1.

### 2.2 Conceptual framework

**Figure 1:**
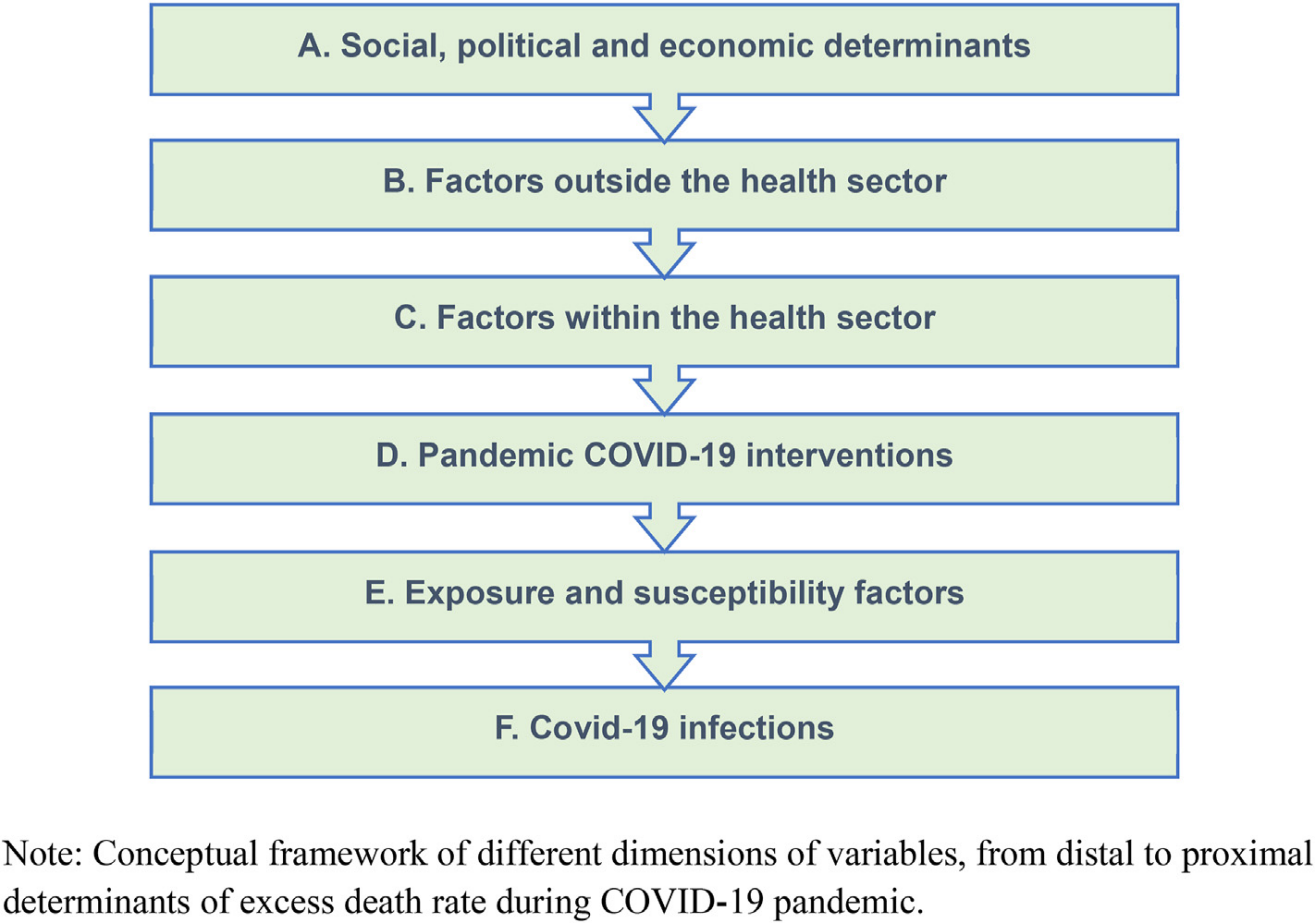
Conceptual framework for COVID-19 and drivers of excess death rate in Peru.

A conceptual framework was developed to guide our analyses, and it considers different hierarchical variables positioned from distal to proximal determinants of excess death rate during the COVID-19 pandemic (Figure 1). We include six dimensions of factors that could explain the excess death rate variation during the study period, namely (*A*) *Social, political and economic determinants:* gross domestic product (GDP) per capita in USD; percentage of poverty; Gini coefficient for income, average per capita monthly income in USD; and percentage of urban population. *(B) Factors outside of the health sector:* unemployment rate, defined as the number of unemployed persons with 14 years of age or more as a percentage of the total number of persons in the labor force (economically active population) (International Labour Organization, 2021); percentage of persons living in overcrowded conditions; percentage of persons with access to improved water sources; percentage of persons with access to improved sanitation facilities; and median years of schooling (Instituto Nacional de Estadistica e Informatica, 2021c). *(C) Factors within the health sector:* percentage of population with public health insurance, government-subsidized health insurance scheme aimed at providing coverage to uninsured citizens (Instituto Nacional de Estadistica e Informatica, 2021c); density of human resources for health, defined as the number of doctors, nurses and midwives per 10,000 population (Ministerio de Salud del Peru, 2021); per capita health expenditure; expenditure per capita of health budget in USD (Ministerio de Economia de Finanzas del Peru, 2021); public healthcare access, defined as the number of outpatient consultations in public health care facilities per 100,000 population (Superintendencia Nacional de Salud de Peru, 2021). *(D) Pandemic related COVID-19 interventions:* per capita expenditure of COVID-19 pandemic budget in USD (Ministerio de Economia de Finanzas del Peru, 2021); and number of COVID-19 tests (PCR molecular tests, serological tests, and antigen rapid tests) per 100,000 population (Presidencia de Consejo de Ministros del Peru, 2021). *(E) Exposure and susceptibility factors:* percentage of people with comorbidities, defined as the percentage of people living with diabetes (physician-diagnosed or diagnosed through fasting glucose test), or hypertension (systolic blood pressure ≥140 mmHg and/or diastolic blood pressure ≥90 mmHg), or obesity (Body mass index ≥30 kg/m^2^)) (Instituto Nacional de Estadistica e Informatica, 2020); percentage of people currently smoking, defined as current tobacco use among persons aged 15 years and older as a percentage of the total persons aged 15 years and older (Instituto Nacional de EstadisticaInformatica, 2020, Instituto Nacional de EstadisticaInformatica, 2021c); and *(F) Covid-19 infections:* COVID-19 incidence, defined as the number of COVID-19 confirmed cases per 100,000 population based on PCR molecular tests, serological tests, or antigen rapid tests (Presidencia de Consejo de Ministros del Peru, 2021); and excess death rate defined as the number of excess deaths per 100,000 population (Feyman et al., 2022; Henry et al., 2022; Islam et al., 2021; Iuliano et al., 2021; Kontis et al., 2020; Kontopantelis et al., 2021; Lewnard et al., 2021; Moghadas and Galvani, 2021; Sempé et al., 2021).

We used the excess death rate as the main indicator of the impact of COVID-19, as it is less prone to underestimation than COVID-19 confirmed deaths only (Feyman et al., 2022; Henry et al., 2022; Islam et al., 2021; Iuliano et al., 2021; Kontis et al., 2020; Kontopantelis et al., 2021; Lewnard et al., 2021; Moghadas and Galvani, 2021; Sempé et al., 2021). We calculated the excess deaths as the difference between the number of deaths from all causes that occurred during January–December 2020 and the expected number of deaths based on a historical baseline, of the previous three years (Feyman et al., 2022; Henry et al., 2022; Islam et al., 2021; Iuliano et al., 2021; Kontis et al., 2020; Kontopantelis et al., 2021; Lewnard et al., 2021; Moghadas and Galvani, 2021; Sempé et al., 2021). In addition to this, we calculated the daily expected deaths using an over-dispersed Poisson model that accounts for temporal trends, seasonal, sex, age and, natural mortality variability by using the *excessmort* package in R (Irizarry Rafael A., 2021), with the following formula:

$$\mu_{s,r,a}\left(t\right)=N_{s,r,a}\left(t\right)\exp\left\{\alpha \left(t\right)+{s\left(t\right)}_{s,r,a}+w\left(t\right)\right\}\;for\;t\in I_{c}$$

Where $\mu_{s,r,a}\left(t\right)$ is the expected number of deaths for individuals of sex (s), in a region (r), and age group (a) at time t, $N_{s,r,a}\left(t\right)$ is the population size, α(t) is a slow trend to account for moving changes in mortality, ${s\left(t\right)}_{s,r,a}$ is a function that accounts for seasonal trends, w(t) is a day of the week effect, and $I_{c}$ is a region specific training time interval that is used to fit the model (Islam et al., 2021). Then, we estimated the rate dividing the excess deaths by the population and multiplying it by 100,000.

### 2.3 Data sources

We used different data sources for gathering the information on variables at regional level. For estimating the excess death rate, we used the National Information System of Deaths, SINADEF (Presidencia de Consejo de Ministros del Peru, 2021). This registry collects information from the entire Peruvian country deaths. It was implemented in 2017 and includes codified information on individual deaths (Ramírez-Soto and Ortega-Cáceres, 2022). GDP per capita was obtained from the National Institute of Statistics and Computing (Instituto Nacional de Estadistica e Informatica, 2021d). Poverty, Gini coefficient, income, unemployment, overcrowding, improved water and sanitation, education and health insurance were obtained from the National Household Surveys (Instituto Nacional de Estadistica e Informatica, 2021b). Data for the National Household Surveys was collected annually in a two stages cluster random sampling approach and urban stratification (Mendoza-Quispe et al., 2021). In the first stage, each cluster contained 120 to 140 household in urban areas and one or more villages together (approximately 120–140 households) in rural areas (Mendoza-Quispe et al., 2021). In the second stage, 10–15 households in the corresponding clusters were selected (Mendoza-Quispe et al., 2021). Trained personnel applied the questionnaires through direct interviews at the households of eligible participants (Mendoza-Quispe et al., 2021). For urban population, comorbidities and smoking, we used information from the National Institute of Statistics and Computing (Instituto Nacional de EstadisticaInformatica, 2021a, Instituto Nacional de EstadisticaInformatica, 2021c). Information on healthcare access was obtained from the SUSALUD (*Superintendencia Nacional de Salud*, in Spanish) website (Superintendencia Nacional de Salud de Peru, 2021). This registry contains information from the entire national health system and collects monthly information about the outpatients consultations in health care facilities. Density of human resources for health was estimated from data provided by the Ministry of Health website (Ministerio de Salud del de Economia de Finanzas del Peru, 2021). Health expenditure and expenditure of COVID-19 pandemic budget were estimated from data available at the Ministry of Economy and Finances (Ministerio de Economia de Finanzas del Peru, 2021). Information on COVID-19 incidence was obtained from the Peruvian Open Source website (Presidencia de Consejo de Ministros del Peru, 2021). This registry contains information from the entire country and collects daily information on COVID-19 cases based on PCR molecular test, serological tests, or antigen rapid tests.

### 2.4 Data analysis

The study period was from January to December 2020 and encompassed four trimesters. All the twenty-five regions were followed across these four periods. Thus, our analysis dataset consisted of 100 observations. For calculating the sample size, we considered the following parameters: alpha = 0.05, power = 0.95, number of groups = 25, number of measurement = 4, effect size 0.2, and a correlation among repeated measurement = 0.3. The resulting sample size was 100. The calculations were done using G∗Power software, version 3.1.9.7 (Faul et al., 2007, 2009). If no information was available by trimester, we considered the most recent information available. Details of the analyses performed are included in the following sections.

### 2.5 Time trends

We described national and regional time trends of the excess death rate and for each variable from our conceptual framework.

### 2.6 National and regional level ranking by excess death rate

We estimated a ranking of regions according to their change of excess death rate over the study period. We additionally contrasted the variation of the excess death rate per region during the trimesters.

### 2.7 Multilevel mixed-effects linear regression

Multilevel mixed-effects linear regressions were run in a stepwise manner, according to a previous report (Huicho et al., 2017). This model considers the fixed effects of the predictors as structured in our conceptual framework, as well as the fixed effects of time. The random effects take into account the variability within regions of Peru over time, as well as between regions. For each box of our proposed conceptual model, starting with box A, we first run crude mixed-effects linear regressions, with excess death rate and one predictor (covariate) at a time. We first selected the variables with p ≤ 0.20, irrespective of their direction, to run afterwards an adjusted multi-level mixed-effects linear regression with time as a locked term. We included time in our models irrespective of the selection criteria to control for the division of the year in four trimesters. Then, we conducted a backward stepwise exclusion of variables with p > 0.20, starting with the variable with the highest p-value. In this way, we obtained the final model for each box, and the variables were incorporated in the final models of the subsequent boxes. We repeated the crude regressions of excess death rate with each predictor in box B as well as the backward stepwise selection for variables with p ≤ 0.20. Then the final selected variables in box B and the final selected variables from box A were run together in a new multivariate model, which was followed by a new backward stepwise selection, to obtain the final model for box A plus box B. The variables of this final model were kept for incorporation in the final models of the next boxes. We repeated the same steps with the next boxes of our conceptual framework, incorporating the selected variables from previous boxes. In the final model, we considered a significance value of p < 0.05.

### 2.8 Oaxaca-Blinder decomposition analysis

For assessing the relative influence of individual predictors on the differences of excess death rate between the trimester with the lowest rate and the trimester with the highest rate, we used the Oaxaca-Blinder decomposition analysis (Jann, 2008). In brief, this statistical approach allows decomposing the change in excess death rate between the selected trimesters into the covariates driving such a change. In terms of model building, variables with a p < 0.20 were retained in the analysis. To avoid multicollinearity, we excluded variables with a high variance inflation factor.

All statistical analyses were conducted with Stata 15.1 (Stata Corp., College Station, TX) and R version 4.1.1 (R Foundation for Statistical Computing). Our study has been reported according to the Strengthening the Reporting of Observational studies in Epidemiology (STROBE) guidelines (Vandenbroucke et al., 2007).

## 2.9 Ethics

Ethical approval of this study was waived by the Ethics Committee of Universidad Peruana Cayetano Heredia (Number: 203713) as it involved the analysis of open access secondary aggregate data from the National Institute of Statistics and Computing and individual-level anonymized data from the National Information System of Deaths.

## 2.10 Role of funding source

This study was funded by researchers’ own funds. All authors had full access to the data in the study. All authors collectively had final responsibility for the decision to submit for publication and guarantee for the data accuracy. The authors alone are responsible for the opinions in the manuscript, which do not necessarily represent those of their institutions.

## 3 Results

### 3.1 Time trends

**Figure 2:**
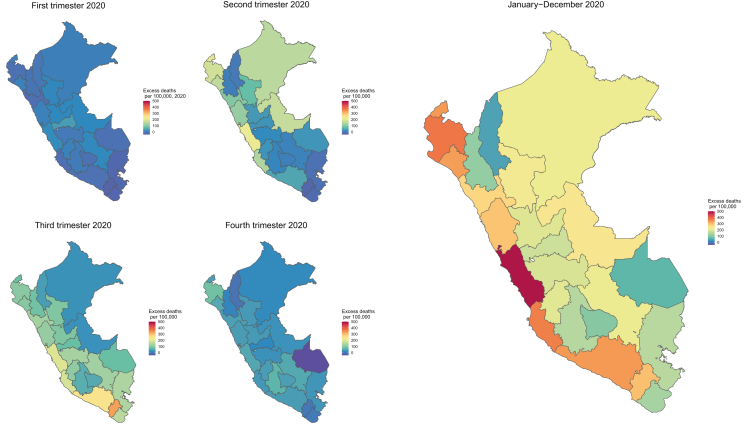
Excess death rate in Peru by trimester and by region, January–December 2000.

The excess death rate showed an increasing trend in all regions, with different slopes. The increase was more evident during the second and third trimesters of 2020 (Table 1 and Supplementary material 2). Excess death rates were higher in the Coast and the northern Amazon during the second trimester 2020, whereas they were higher in the southern Coast and in the Highlands during the third trimester 2020. Arequipa and Moquegua (regions predominantly coastal) showed the highest excess deaths rates in the third trimester 2020, being also the highest excess death rates per trimester during the whole study period (Figure 2).

**Table 1:** Excess death rate in Peru, by region and trimester: January–December 2020.

Region	Whole period		Trimester 1		Trimester 2		Trimester 3		Trimester 4	
	Excess death rate∗	Ranking	Excess death rate∗	Ranking	Excess death rate∗	Ranking	Excess death rate∗	Ranking	Excess death rate∗	Ranking
PERU	329.1	-	10.5	-	128.0	-	145.7	-	44.8	-
LIMA	482.6	1	24.1	2	211.2	2	195.0	3	52.3	1
CALLAO	479.8	2	11.6	10	256.8	1	168.4	5	43.0	2
PIURA	368.5	3	-5.9	20	179.8	4	107.8	13	86.8	3
ICA	360.5	4	-6.8	21	143.4	8	186.3	4	37.5	4
TUMBES	336.6	6	8.0	12	203.5	3	81.7	18	43.4	5
AREQUIPA	336.6	5	-5.3	19	50.7	12	249.7	2	41.5	6
LAMBAYEQUE	332.0	7	12.0	9	160.3	6	105.1	14	54.6	7
MOQUEGUA	296.5	8	-18.0	25	-16.8	25	329.2	1	2.1	8
ANCASH	291.2	9	12.8	7	118.0	9	118.6	11	41.7	9
LA LIBERTAD	271.3	10	-7.8	22	103.0	10	131.3	9	44.7	10
UCAYALI	246.2	11	20.6	4	167.0	5	28.6	25	30.0	11
SAN MARTIN	234.2	12	19.3	6	76.7	11	100.2	15	38.0	12
LORETO	219.0	13	10.7	11	156.9	7	29.7	24	21.7	13
CUSCO	218.4	14	21.4	3	13.0	19	130.7	10	53.3	14
HUANCAVELICA	198.8	15	12.8	8	31.6	16	112.1	12	42.2	15
HUANUCO	195.4	16	20.4	5	36.6	13	99.6	16	38.9	16
JUNIN	189.3	17	4.9	14	22.3	17	133.1	8	29.0	17
PASCO	173.7	18	41.5	1	33.3	14	79.1	20	19.8	18
PUNO	152.8	19	-14.1	23	-6.3	23	140.0	7	33.1	19
AYACUCHO	150.7	20	7.0	13	17.3	18	59.6	21	66.7	20
TACNA	135.1	21	-16.1	24	-11.0	24	156.9	6	5.3	21
CAJAMARCA	115.3	22	0.3	16	10.4	21	87.8	17	16.7	22
APURIMAC	104.9	23	-4.8	17	11.3	20	55.1	22	43.2	23
MADRE DE DIOS	68.5	24	-4.9	18	33.0	15	80.4	19	-40.0	24
AMAZONAS	46.8	25	1.6	15	4.4	22	42.3	23	-1.5	25

∗ Number of excess deaths per 100,000 population.

Time trends of the different study variables are shown in Supplementary material 2. The majority of variables in Box A showed a worsening trend, for example GDP per capita and per capita income decreased over time, while poverty and Gini coefficient increased in the majority of regions. Urban population remained the same in major regions like Lima and Callao, while some variability was found in other regions. In Box B, access to an improved water and sanitation showed a flat trend, while schooling decreased and unemployment increased substantially with a small reduction in the last trimester, and overcrowding conditions did not show a defined change. In Box C, per capita health expenditure showed a substantial increase, while health insurance and density of human resources for health showed a slight increase in the majority of regions. In contrast, healthcare access showed a large decrease, with a small recovery at the end of the year. In Box D, per capita expenditure of COVID-19 pandemic health budget showed a large increase per trimester, while the number of COVID-19 test showed a large increase in the second and third trimesters, with a considerable decrease in the fourth trimester. In Box E, the percentage of people with comorbidities and the percentage of smoking people decreased slightly in the majority of regions. In Box F, COVID-19 incidence showed a large increase, with a more prominent peak in the third trimester.

### 3.2 Regional ranking by excess death rate

The national excess death rate for year 2020 was 329.1 per 100,000 population, with a peak reached on the third trimester (145.7). At regional level, Lima (482.6), Callao (479.8) and Piura (368.5), all coastal regions, showed the highest excess death rates. Apurimac (104.9), Madre de Dios (68.5) and Amazonas (46.8), the first one a Highlands region and the last two ones Amazon regions, showed the lowest excess death rates. In the third trimester, Moquegua (329.2), Arequipa (249.7) and Lima (195.0) showed the highest excess death rates (Table 1).

### 3.3 Multilevel mixed-effects linear regression model

The time-adjusted model shows that various factors were significantly associated with the excess death rate including urbanization, unemployment, access to improved water sources, access to improved sanitation facilities, years of schooling, health insurance, per capita health expenditure, healthcare access, per capita expenditure of COVID-19 pandemic health budget, number of COVID-19 test, comorbidities, and COVID-19 Incidence (Table 2). After adjustment for time and confounders, only the association between healthcare access and COVID-19 incidence remained (Table 2).

**Table 2:** Multilevel linear models for excess death rate.

Dimension of the predictor variables	Predictor variables	Time-adjusted regression coefficient	95% CI	p-value	Time- and confounder-adjusted regression coefficient	95% CI	p-value
	Time (phase)	12.392	(0.422, 24.362)	0.042	-9.040	(-16.971, -1.108)	0.025
Box A. Social, political and economic determinants	GDP per capita (thousand USD)	4.736	(-1.644, 11.116)	0.146	-	-	-
	Poverty (%)	-0.913	(-2.139, 0.313)	0.144	-	-	-
	Gini coefficient for income	-148.271	(-451.733, 155.192)	0.338	-	-	-
	Average per capita monthly income (USD)	0.215	(-0.021, 0.452)	0.074	-	-	-
	Urban population (%)	0.876	(0.249, 1.502)	0.006	-	-	-
Box B. Factors outside the health sector	Unemployment rate (%)	8.443	(5.506, 11.380)	<0.001	-	-	-
	Overcrowded conditions (%)	-3.104	(-6.385, 0.177)	0.064	-	-	-
	Access to improved water sources (%)	0.665	(0.082, 1.249)	0.025	-	-	-
	Access to improved sanitation facilities (%)	0.939	(0.205, 1.673)	0.012	-	-	-
	Years of schooling (median)	9.216	(2.140, 16.292)	0.011	-	-	-
Box C. Factors within the health sector	Health insurance (%)	-1.284	(-2.137, -0.431)	0.003	-	-	-
	Density of human resources for health (per 10,000 population)	-1.518	(-3.426, 0.389)	0.119	-	-	-
	Per capita health expenditure (USD)	-1.342	(-2.246, -0.439)	0.004	-	-	-
	Healthcare access (per 100,000 population)	-0.006	(-0.008, -0.004)	<0.001	-0.004	(-0.005, -0.002)	<0.001
Box D. Pandemic COVID-19 interventions	Per capita expenditure of COVID-19 pandemic health budget (USD)	-1.204	(-1.905, -0.502)	0.001	-	-	-
	Number of COVID-19 test (per 100,000 population)	0.059	(0.005, 0.007)	<0.001	-	-	-
Box E. Exposure and susceptibility factors	People with comorbidities (%)	2.052	(0.179, 3.925)	0.032	-	-	-
	People currently smoking (%)	0.927	(-2.895, 4.749)	0.635	-	-	-
Box F. Covid-19 infections	COVID-19 Incidence (per 100,000 population)	0.062	(0.051, 0.072)	<0.001	0.052	(0.042, 0.063)	<0.001

Values represent time-adjusted coefficients and 95% coefficient interval (CI). Comorbidities included hypertension, diabetes mellitus and obesity.

### 3.4 Oaxaca-Blinder decomposition analysis

**Figure 3:**
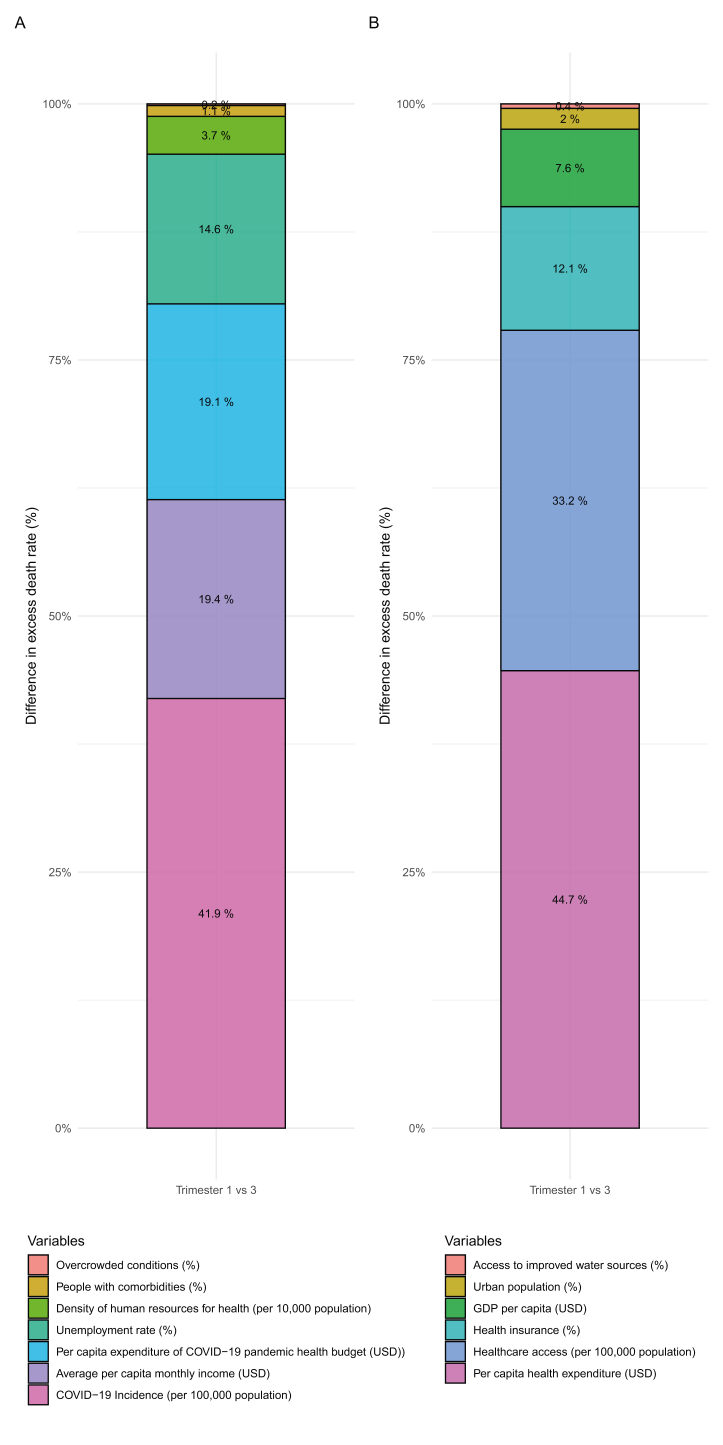
Oaxaca blinder decomposition explaining differences in excess death rate between trimester 1 and trimester 3. Decomposition analysis showing the relative contribution to difference in excess death rate between trimester 1 and trimester 3. (A) Decomposition analysis showing factors with positive contribution (risk factors) and (B) factors with negative contribution (protective factors) to the relative difference in excess death rate.

Figure 3 shows the relative contribution of each variable to the excess death rate difference between the trimester with the lowest excess death rate and the trimester with the highest rate (trimesters 1 and 3, respectively). Risk factors included COVID-19 incidence (41.9%), per capita income (19.4%), COVID-19 budget expenditure (19.1%), unemployment rate (14.6%), density of human resources for health (3.7%), comorbidities (1.1%), and overcrowding (0.2%) (Figure 3A). Protective factors included per capita health expenditure (44.7%), healthcare access (33.2%), health insurance (12.1%), GDP per capita (7.6%), urban population (2.0%), and access to improved water sources (0.4%) (Figure 3B).

## 4 Discussion

This study shows the factors associated with excess death rate during the COVID-19 pandemic in Peru in 2020 and the relative contribution of individual factors to the excess death rate. Our multilevel model showed that higher healthcare access and higher COVID-19 incidence were associated with lower and higher excess death rates, respectively. When comparing the trimesters with the lowest and the highest excess death rates, our decomposition analysis showed COVID-19 incidence, per capita income and unemployment rate as the main risk factors, while the main protective factors were per capita health expenditure, healthcare access and health insurance.

Our study showed that a lower healthcare access was associated with a higher excess death rate over time. The COVID-19 pandemic has underscored the barriers in healthcare access and social vulnerabilities that arise from a fragmented healthcare system in Peru. Our finding is concordant with previous studies (Hernández-Vásquez et al., 2021; Pilkington et al., 2021). However, to best of our knowledge, no previous literature has focused systematically on the drivers of excess death rate during COVID-19 over time. A study in France among 119,546 persons, using a multiscale geographically weighted regression, reported that the excess all-cause mortality was significantly lower in departments where the supply of primary healthcare providers was higher (Pilkington et al., 2021). Likewise, an ecological study in Peru reported that more than half of the persons with COVID-19 symptoms –with and without health insurance— did not use health services because of geographical and socioeconomic barriers (Hernández-Vásquez et al., 2021). The COVID-19 pandemic found a country with persistent health, social and economic inequities characterized by low healthcare access. In fact, healthcare access is a comprehensive proxy to understand the factors producing inequalities at population level. In addition to this, our study showed that higher COVID-19 incidence was associated with higher excess death rate over time, which is concordant with previous findings in Peru and in other countries (Alimohamadi et al., 2021; Kazemi-Karyani et al., 2020; Konstantinoudis et al., 2022; Piroth et al., 2021; Ramírez-Soto and Ortega-Cáceres, 2022; Ramírez-Soto et al., 2022). Besides, case fatality rate of COVID-19 may not be similar across countries (Alimohamadi et al., 2021; Kazemi-Karyani et al., 2020). This variability in the fatality rate of COVID-19 may be explained by differences in the sociodemographic characteristics, health and social care systems (Alimohamadi et al., 2021; Kontis et al., 2020).

Our decomposition analysis also reveals that per capita monthly income, unemployment rate, people with comorbidities, and overcrowded conditions were risk factors associated with a higher excess death rate. Consistent with our findings, a nationwide analysis at the county level conducted in the United States (US) found that higher COVID-19 mortality was associated with higher income ratio but lower education (Hawkins et al., 2020). Moreover, unemployment has been associated with an increased mortality risk before and during the COVID-19 pandemic (Brenner M. Harvey, 2021; Nie et al., 2020; Roelfs et al., 2011). Another study in the US found that ten percent of unemployment was associated with 48,149 excess deaths from February to November 2020, which was more evident in ethnic minorities (Brenner M. Harvey, 2021). Similarly, temporary or long-term unemployed participants had an increased risk of mortality from all causes than employed participants in the US (Nie et al., 2020). A meta-analysis among 20 million persons found an association between unemployment and all-cause mortality among working-age persons, particularly for those in their early and middle careers (Roelfs et al., 2011). Our study also suggests that unemployment might have played a significant role in the excess death during COVID-19 pandemic. COVID-19 might have also exacerbated not only health inequities, but also economic and social inequalities at the population level.

Our decomposition analysis identified comorbidities as a risk factor, although with a small effect on the excess death rate. Increasing evidence shows that COVID-19 patients with comorbidities have the worst prognosis (Holman et al., 2020; Zhu et al., 2020). A retrospective study in China among 7,337 patients with COVID-19 and type 2 diabetes found that subjects with type 2 diabetes had a higher mortality hazard ratio than the non-diabetic individuals (Zhu et al., 2020). Another study in China among 1,833 patients with COVID-19 found that patients with hypertension had a higher mortality risk (Mubarik et al., 2021). A meta-analysis among 625,153 patients found that obesity was associated with higher COVID-19 mortality when compared with non-obese patients (Cai et al., 2021). Likewise, our decomposition analysis showed overcrowding as a risk factor, although with minor effects. Poor housing has been identified as a factor that increases the risk of COVID-19 mortality (Ahmad et al., 2020; Reyes-Vega et al., 2021). An observational study in the US among 3,123 counties found that counties with high rates of people living in overcrowding conditions have higher COVID-19 deaths (Ahmad et al., 2020). People living in overcrowded and medically underserved conditions are generally vulnerable groups who also have more comorbidities and thus a greater need for healthcare access.

Despite previous studies reporting that lower density of human resources increased the risk of excess death rates during the COVID-19 pandemic (Kapitsinis, 2021). In our study, we found that higher density of human resources and higher COVID-19 pandemic budget expenditure were associated with higher excess death rates. We cautiously speculate that this might reflect the effect of overwhelmed health facilities during the pandemic, which might have overcome the health workforce and the expenditure efforts (Kapitsinis, 2021).

Similar with our results, previous studies have reported that higher GDP per capita and urbanization are protective factors that decreased the excess death rate (Coccia, 2021; Yu et al., 2021). A previous global study found that higher GDP per capita is associated with a reduction of fatality rate of COVID-19 between countries (Coccia, 2021). In addition, an observational study on 32 Chinese provinces found that 1% increase in urbanization reduced the risk of COVID-19 mortality by 0.2% (Yu et al., 2021). In line with other reports (Abedi et al., 2021; Ji et al., 2020; Liang et al., 2020; Pilkington et al., 2021; Scannell Bryan et al., 2021; Xie et al., 2021), our decomposition analysis also showed that health expenditure, health insurance and healthcare access were all protective factors of the excess death rate, highlighting the importance of health resources availability during the pandemic (Abedi et al., 2021; Ji et al., 2020; Liang et al., 2020; Pilkington et al., 2021; Scannell Bryan et al., 2021; Xie et al., 2021).

Multiple factors have been recognized to influence the differences in excess deaths between countries during the COVID-19 pandemic (Kapitsinis, 2021). Growing evidence suggest that COVID-19 pandemic has disproportionally affected the poor and the minorities. This was evident in Latin American countries like Chile and Brazil (de Souza et al., 2020; Mena et al., 2021). In Peru, our results showed a deterioration of various socioeconomic and health indicators during 2020 in all regions (e.g., increase of unemployment rate and decrease of healthcare access). Factors such GDP per capita reflect a country's socioeconomic performance, which is associated with better health. People living in countries with lower GDP per capita might have poor access to health services, resulting in poor health (Pardhan and Drydakis, 2021).

The association of higher per capita income with higher excess death rates in our analysis might reflect the influence of the higher number of vulnerable employments, which is estimated in more than 50% in Peru, on the number of deaths during the pandemic (Cifuentes-Faura, 2021; World Bank Data, 2019). Differences in living conditions between urban and rural areas might also contribute to reduce the incidence and mortality of COVID-19. Thus, better levels of housing, health and social services in urban areas might have a positive effect on the prevention and control of infectious diseases (Yu et al., 2021). Health consequences of unemployment are well recognized in previous studies, based on the economic deprivation model and stress theory (Burgard et al., 2007; Hammarström and Janlert, 2002; Muntaner et al., 2010). The economic deprivation model explains how the deterioration of the economic position and job benefits undermine health (Muntaner et al., 2010). Unemployment is also a stressor that may lead to health-related behaviors and physiological changes including impairment of the immune system (Muntaner et al., 2010). Consequently, this could increase the risk of morbidity and mortality during the pandemic. Moreover, an increased number of COVID-19 infections due to poor house conditions might be responsible of the increased excess death rate in overcrowded houses without access to sanitation facilities (Pardhan and Drydakis, 2021; Reyes-Vega et al., 2021). On the other hand, the effect of higher education on lower COVID-19 mortality might be mediated by decreased prevalence of smoking, physical activity and obesity, as observed in different studies (Hoeymans et al., 1996; Yoshikawa and Asaba, 2021). It is also known that well-funded and supported healthcare systems can contribute to effectively tackling a pandemic (Kapitsinis, 2021). By contrast, an overwhelmed health system with inadequate number of doctors, beds, health funds, during circumstances like the COVID-19 pandemic can result in higher excess death rates (Kapitsinis, 2021).

The COVID-19 pandemic has exposed the long-lasting social, economic and health inequities at national and regional level in Peru. Sound policies and interventions are needed to tackle social, economic and health inequities and to safeguard those most at risk, preventing them from needless and avoidable suffering and death in the current pandemic and in future health crises.

### 4.1 Strengths and limitations

Our study is unique in trying to systematically disentangle the role of different factors that would influence the excess death rate attributable to Covid-19 in Peru through a combination of methodological approaches, including a confounding-adjusted multilevel regression analysis and a decomposition analysis. We used a comprehensive quantitative approach that included different predictors from distal to proximal. We acknowledge some limitations. First, the excess death rate is based on a short reference period from 2017-2019. However, the number of deaths in a given year depend on multiple factors that affect survival, and previous studies have shown that it is appropriate to select a period of time, which is closer in time to the observed period (Dorrucci et al., 2021). Second, our socioeconomic multilevel analysis is prone to ecological fallacy and might not necessarily reflect what would happen at individual level. However, multilevel analyses are useful to highlight the country inequities and their distribution and to generate further hypotheses in the absence of individual level data (de Souza et al., 2020). Finally, there is the possibility that additional adjustment could be needed to better capture the complex interactions of variables from the different dimensions that may influence the excess death rates.

## 5 Conclusion

Our study suggests that the drastic increase of the excess death rate during the COVID-19 pandemic in Peru may have been influenced by other factors besides COVID-19 incidence, from distal to proximal drivers, including socioeconomic determinants, factors outside and within the health sector, and susceptibility factors. Further studies at individual level are needed to corroborate our findings.

## Declarations

### Author contribution statement

Kim N. Cajachagua-Torres: Conceived and designed the experiments; Performed the experiments; Analyzed and interpreted the data; Contributed reagents, materials, analysis tools or data; Wrote the paper.

Hugo G. Quezada-Pinedo: Performed the experiments; Analyzed and interpreted the data; Contributed reagents, materials, analysis tools or data; Wrote the paper.

Carlos A Huayanay-Espinoza and Jordan A Obeso-Manrique: Analyzed and interpreted the data; Contributed reagents, materials, analysis tools or data.

Víctor A Peña-Rodríguez and Elisa Vidal: Analyzed and interpreted the data.

Luis Huicho: Conceived and designed the experiments; Analyzed and interpreted the data; Contributed reagents, materials, analysis tools or data; Wrote the paper.

### Funding statement

This research did not receive any specific grant from funding agencies in the public, commercial, or not-for-profit sectors.

### Data availability statement

Data associated with this study has been deposited at

https://www.datosabiertos.gob.pe/

https://www.inei.gob.pe/bases-de-datos/

### Declaration of interests statement

The authors declare no conflict of interest.

### Additional information

No additional information is available for this paper.
