# Supplementary material for: COVID-19 and drivers of excess death rate in Peru: A longitudinal ecological study
Source: Heliyon. 2022 Nov 30;8(12):e11948. doi: 10.1016/j.heliyon.2022.e11948 (PMC9710104; doi:10.1016/j.heliyon.2022.e11948)
Supplement: Supplementary material.pdf [file mmc1.pdf]

Supplementary material

COVID-19 and drivers of excess death rate in Peru: A longitudinal ecological study

Short title: *COVID-19 and drivers of excess death rate in Peru*

Kim N Cajachagua-Torres[1,2], Hugo G Quezada-Pinedo[1,2], Carlos A Huayanay-Espinoza[2], Jordan A Obeso-Manrique[2], Víctor A Peña-Rodríguez[3,4], Elisa Vidal[2], Luis Huicho[2,5]

[1] The Generation R Study Group, Erasmus MC, University Medical Centre Rotterdam, 3000 CA, Rotterdam, The Netherlands.

[2] Centro de Investigación en Salud Materna e Infantil and Centro de Investigación para el Desarrollo Integral y Sostenible, Universidad Peruana Cayetano Heredia, 150135, Lima, Peru.

[3] Facultad de Ciencias Físicas, Universidad Nacional Mayor de San Marcos, 15081, Lima, Peru.

[4] Centro de Investigaciones Tecnológicas, Biomédicas y Medioambientales, 15081, Lima, Peru.

[5] Facultad de Medicina "Alberto Hurtado", Universidad Peruana Cayetano Heredia, 150135, Lima, Peru

Corresponding author: Kim N Cajachagua-Torres**,** MD. The Generation R Study, Erasmus MC, University Medical Centre Rotterdam, Rotterdam, The Netherlands, P.O. Box 2040, 3000 CA Rotterdam, the Netherlands. Email: k.cajachaguatorres@erasmusmc.nl

CONTENTS

Supplementary material 1. Principal norms during 2020

Supplementary material 2. Departmental time trends of variables of the conceptual framework

Supplementary material 3. Strengthening the Reporting of Observational studies in Epidemiology (STROBE) checklist

Supplementary material 1. Principal norms during 2020

Date	Major government interventions	Comments
15/03/2020	Decreto Supremo N° 044-2020-PCM, Decreto Supremo que declara Estado de Emergencia Nacional por las graves circunstancias que afectan la vida de la Nación a consecuencia del brote del COVID-19 (Presidencia del Consejo de Ministros (PCM), 2020a).	Norm related to the restrictions to people to leave their home and travel freely during the pandemic
23/05/2020	Decreto Supremo Nº 094-2020-PCM, Decreto Supremo que establece las medidas que debe observar la ciudadanía hacia una nueva convivencia social y prorroga el Estado de Emergencia Nacional por las graves circunstancias que afectan la vida de la Nación a consecuencia del COVID-19 (Presidencia del Consejo de Ministros (PCM), 2020b).	Extensions to the Decreto Supremo, Nº 044-2020-PCM norm related to the restrictions to people to leave their home and travel freely during the pandemic.
02/05/2020	Decreto Supremo Nº 080-2020-PCM, Decreto Supremo que aprueba la reanudación de actividades económicas en forma gradual y progresiva dentro del marco de la declaratoria de Emergencia Sanitaria Nacional por las graves circunstancias que afectan la vida de la Nación a consecuencia del COVID-19. (Presidencia del Consejo de Ministros (PCM), 2020e).	Norm related to the first economic reactivation. Include large scale mining projects, transportation, sanitation, e-commerce, restaurant by delivery, etc.
04/06/2020	Decreto Supremo Nº 101-2020-PCM, Decreto Supremo que aprueba la Fase 2 de la Reanudación de Actividades Económicas dentro del marco de la declaratoria de Emergencia Sanitaria Nacional por las graves circunstancias que afectan la vida de la Nación a consecuencia del COVID-19, y modifica el Decreto Supremo Nº 080-2020-PCM (Presidencia del Consejo de Ministros (PCM), 2020g).	Norm related to the second economic reactivation. Included medium scale mining project, studies of environmental impact, project of public and private investment, wine, tobacco, alcohol industries, footwear sector, restaurants, malls and hotels under reduced capacity, national transportation for authorized activities, etc.
26/06/2020	Decreto Supremo N° 116-2020-PCM, Decreto Supremo que establece las medidas que debe observar la ciudadanía en la Nueva Convivencia Social y prorroga el Estado de Emergencia Nacional por las graves circunstancias que afectan la vida de la Nación a consecuencia del COVID-19 (Presidencia del Consejo de Ministros (PCM), 2020c).	Norm related to partial lockdown for the following regions: Arequipa, Ica, Junín, Huánuco, San Martín, Madre de Dios y Ancash.
30/06/2020	Decreto Supremo Nº 117-2020-PCM, Decreto Supremo que aprueba la Fase 3 de la Reanudación de Actividades Económicas dentro del marco de la declaratoria de emergencia sanitaria nacional por las graves circunstancias que afectan la vida de la Nación a consecuencia del COVID-19 (Presidencia del Consejo de Ministros (PCM), 2020d).	Norm related to the third economic reactivation. Included all remaining activities in mining and agriculture. Restaurants at 40% of capacity. Stores at 50% of capacity, national flight, etc.
25/09/2020	Decreto Supremo Nº 157-2020-PCM, Decreto Supremo que aprueba la Fase 4 de la reanudación de actividades económicas dentro del marco de la declaratoria de Emergencia Sanitaria Nacional por las graves circunstancias que afectan la vida de la Nación a consecuencia del COVID-19 (Presidencia del Consejo de Ministros (PCM), 2020f).	Norm related to the fourth economic reactivation. Restaurants at 60% of capacity, stores at 60% of capacity, sea and river transport at 50% of capacity, international flight transportation, etc.

References – Supplementary material 1

Presidencia del Consejo de Ministros (PCM) (2020a). Decreto Supremo N° 044-2020-PCM, Decreto Supremo que declara Estado de Emergencia Nacional por las graves circunstancias que afectan la vida de la Nación a consecuencia del brote del COVID-19.

Presidencia del Consejo de Ministros (PCM) (2020b). Decreto Supremo N° 094-2020-PCM, Decreto Supremo que establece las medidas que debe observar la ciudadanía hacia una nueva convivencia social y prorroga el Estado de Emergencia Nacional por las graves circunstancias que afectan la vida de la Nación a consecuencia del COVID-19.

Presidencia del Consejo de Ministros (PCM) (2020c). DECRETO SUPREMO N° 116-2020-PCM, Decreto Supremo que establece las medidas que debe observar la ciudadanía en la Nueva Convivencia Social y prorroga el Estado de Emergencia Nacional por las graves circunstancias que afectan la vida de la Nación a consecuencia del COVID-19.

Presidencia del Consejo de Ministros (PCM) (2020d). Decreto Supremo N° 117-2020-PCM, Decreto Supremo que aprueba la Fase 3 de la Reanudación de Actividades Económicas dentro del marco de la declaratoria de emergencia sanitaria nacional por las graves circunstancias que afectan la vida de la Nación a consecuencia del COVID-19.

Presidencia del Consejo de Ministros (PCM) (2020e). Decreto Supremo Nº 080-2020-PCM, Decreto Supremo que aprueba la reanudación de actividades económicas en forma gradual y progresiva dentro del marco de la declaratoria de Emergencia Sanitaria Nacional por las graves circunstancias que afectan la vida de la Nación a consecuencia del COVID-19.

Presidencia del Consejo de Ministros (PCM) (2020f). DECRETO SUPREMO Nº 157-2020-PCM, Decreto Supremo que aprueba la Fase 4 de la reanudación de actividades económicas dentro del marco de la declaratoria de Emergencia Sanitaria Nacional por las graves circunstancias que afectan la vida de la Nación a consecuencia del COVID-19.

Presidencia del Consejo de Ministros (PCM) (2020g). Decreto Supremo Nº 101-2020-PCM, Decreto Supremo que aprueba la Fase 2 de la Reanudación de Actividades Económicas dentro del marco de la declaratoria de Emergencia Sanitaria Nacional por las graves circunstancias que afectan la vida de la Nación a consecuencia del COVID-19, y modifica el Decreto Supremo Nº 080-2020-PCM.

Supplementary material 2

GDP per capita (thousands USD) by region

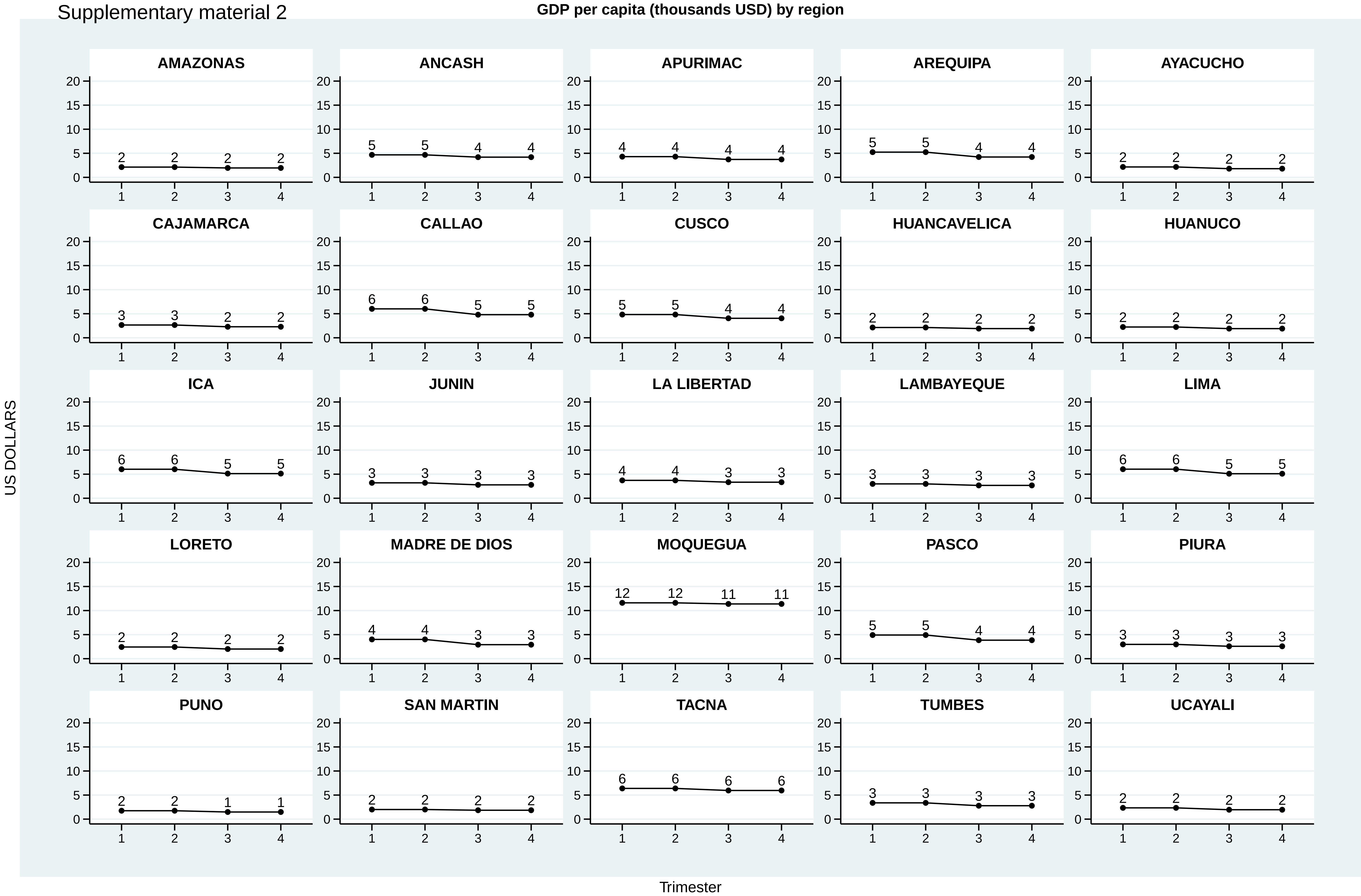

Poverty (%) by region

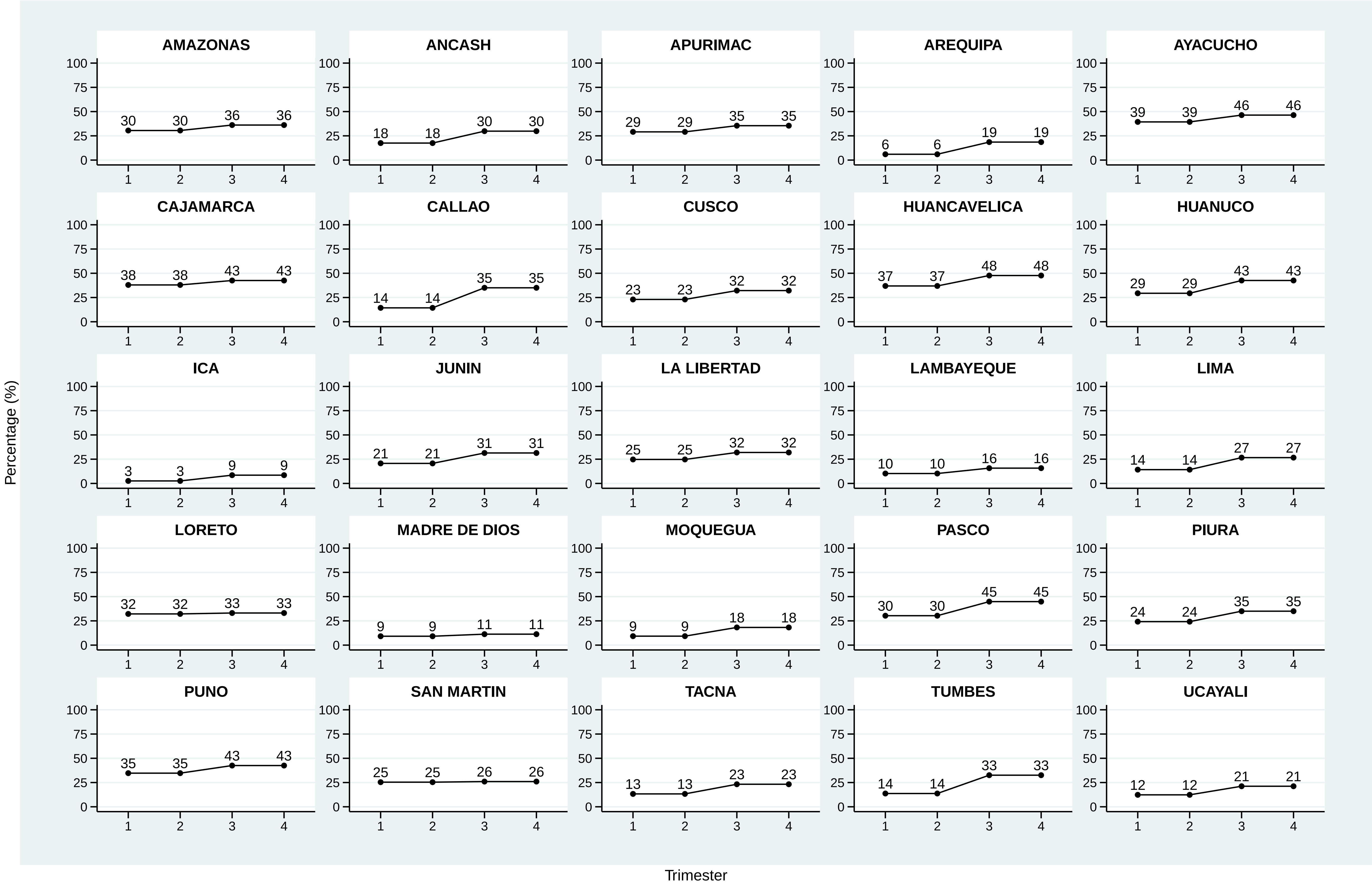

Gini coefficient for income by region

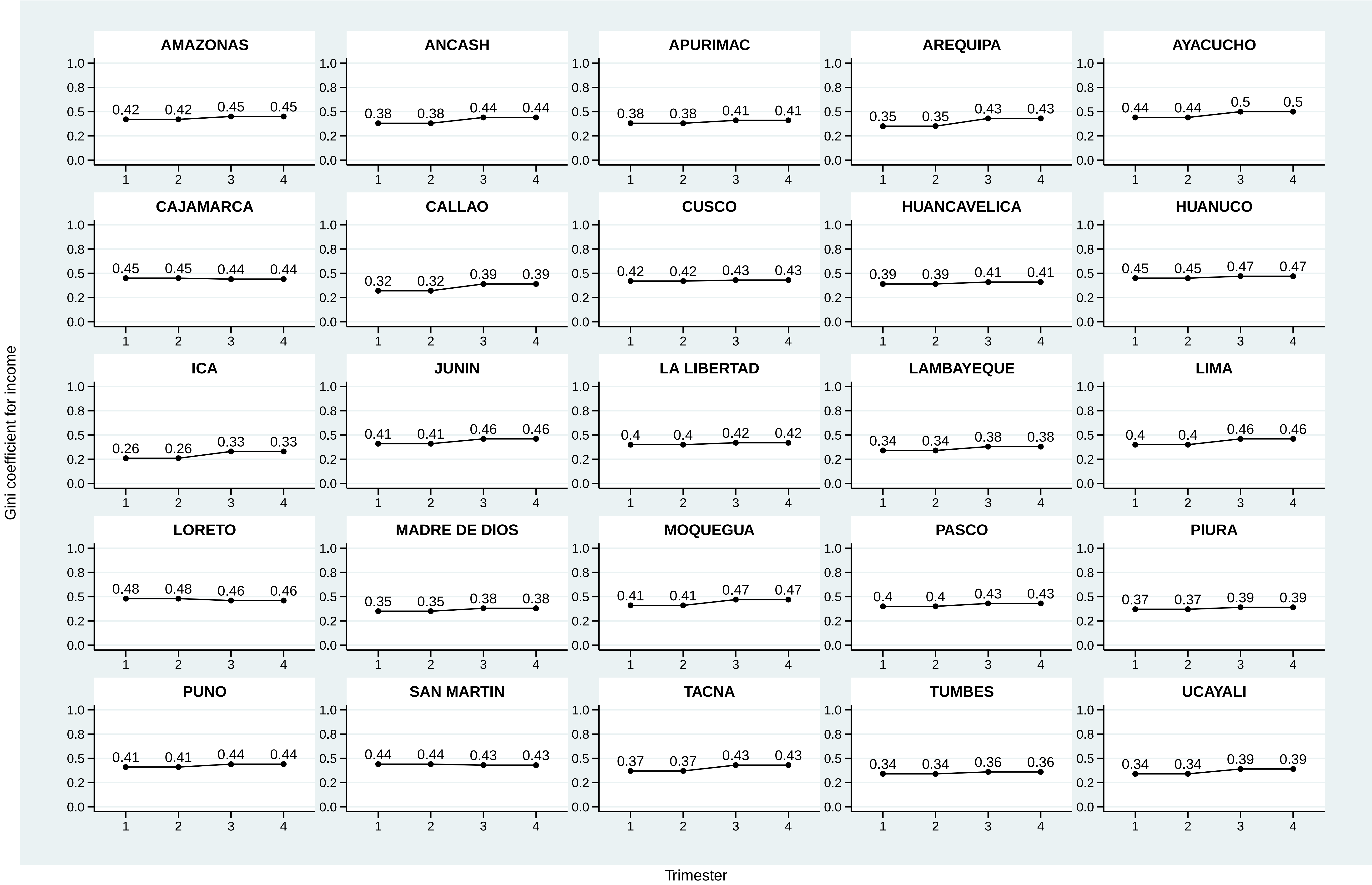

Average per capita monthly income (USD) by region

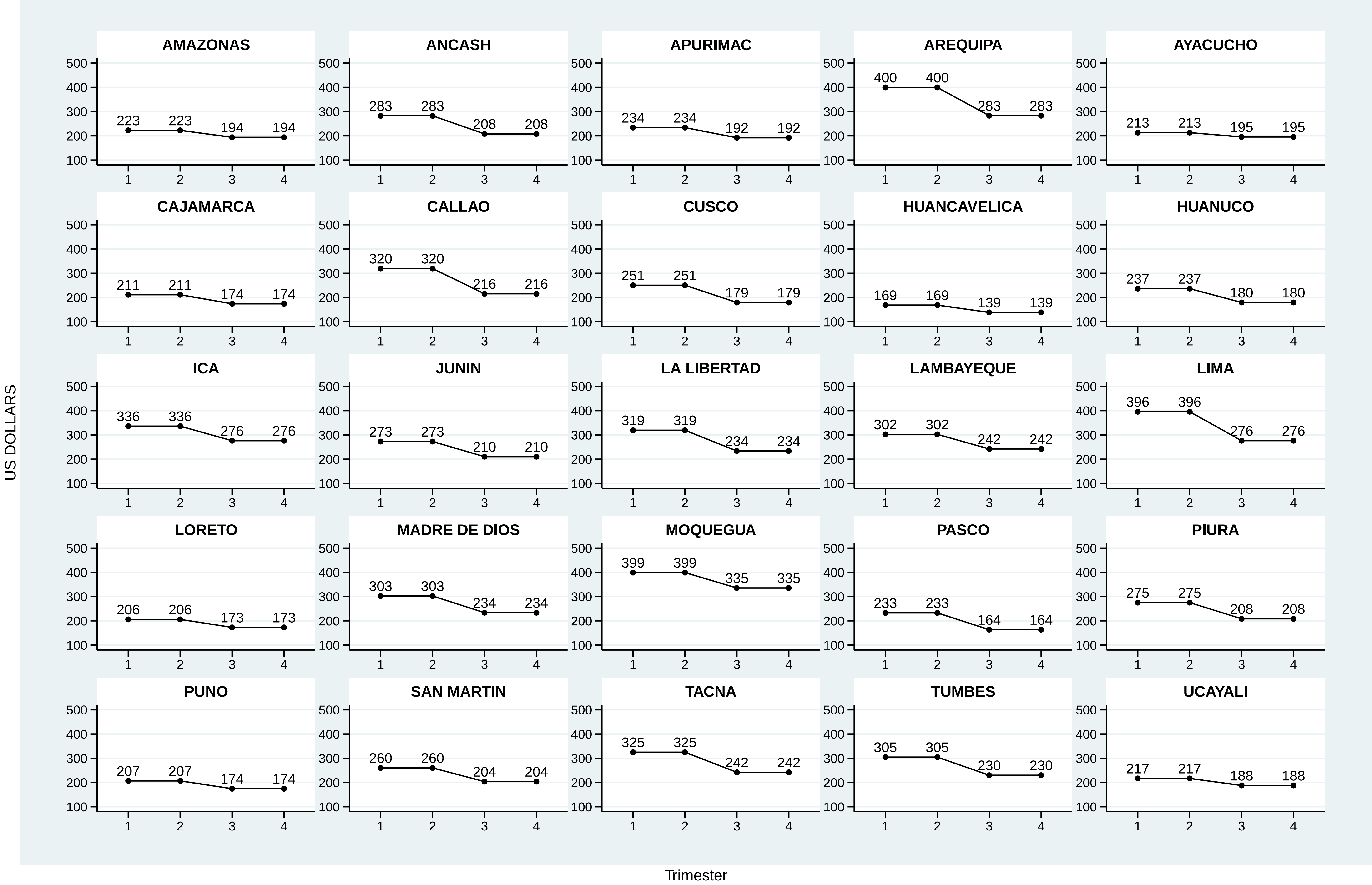

Urban population (%) by region

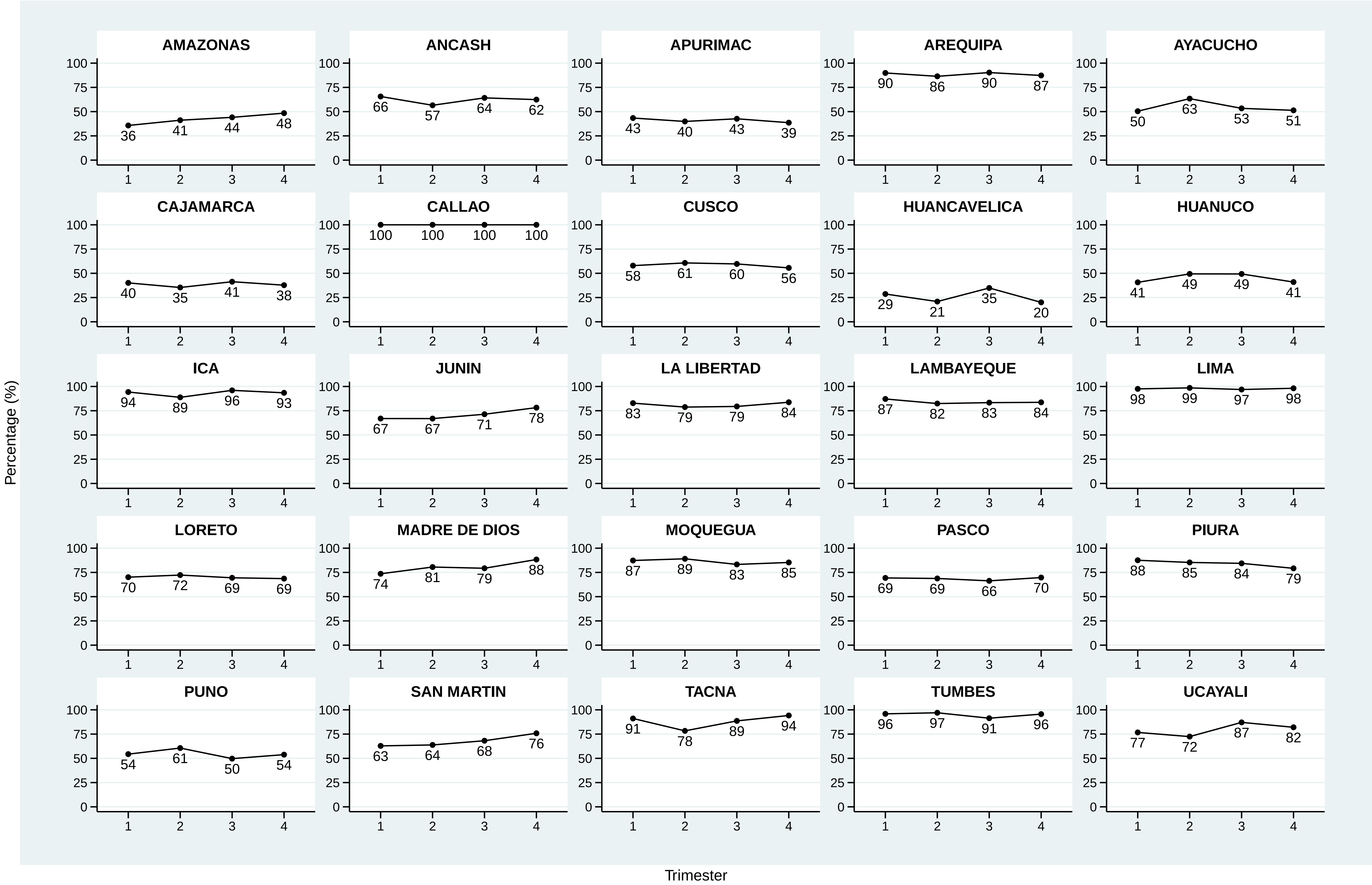

Unemployment rate (%) by region

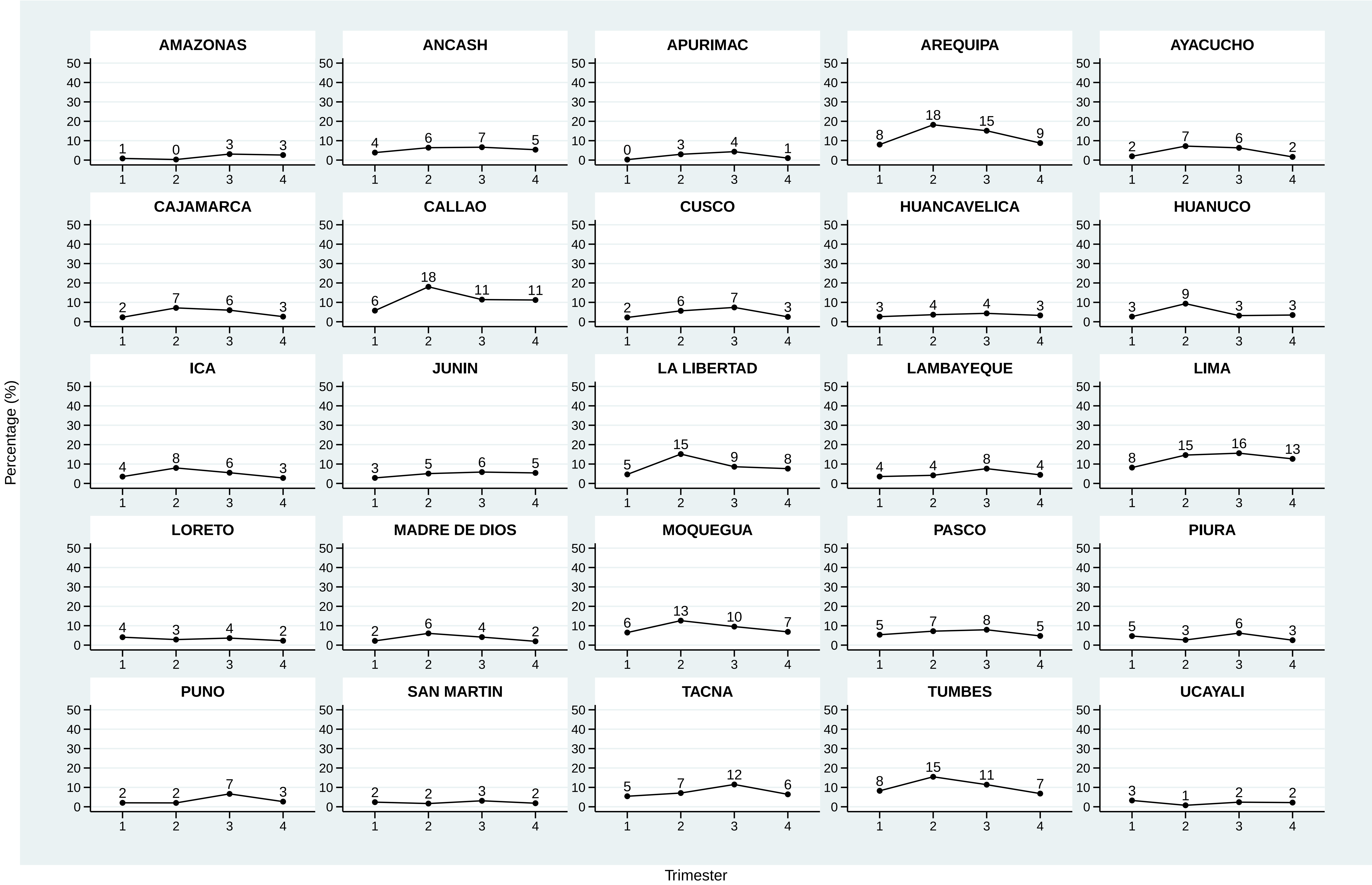

Overcrowded conditions (%) by region

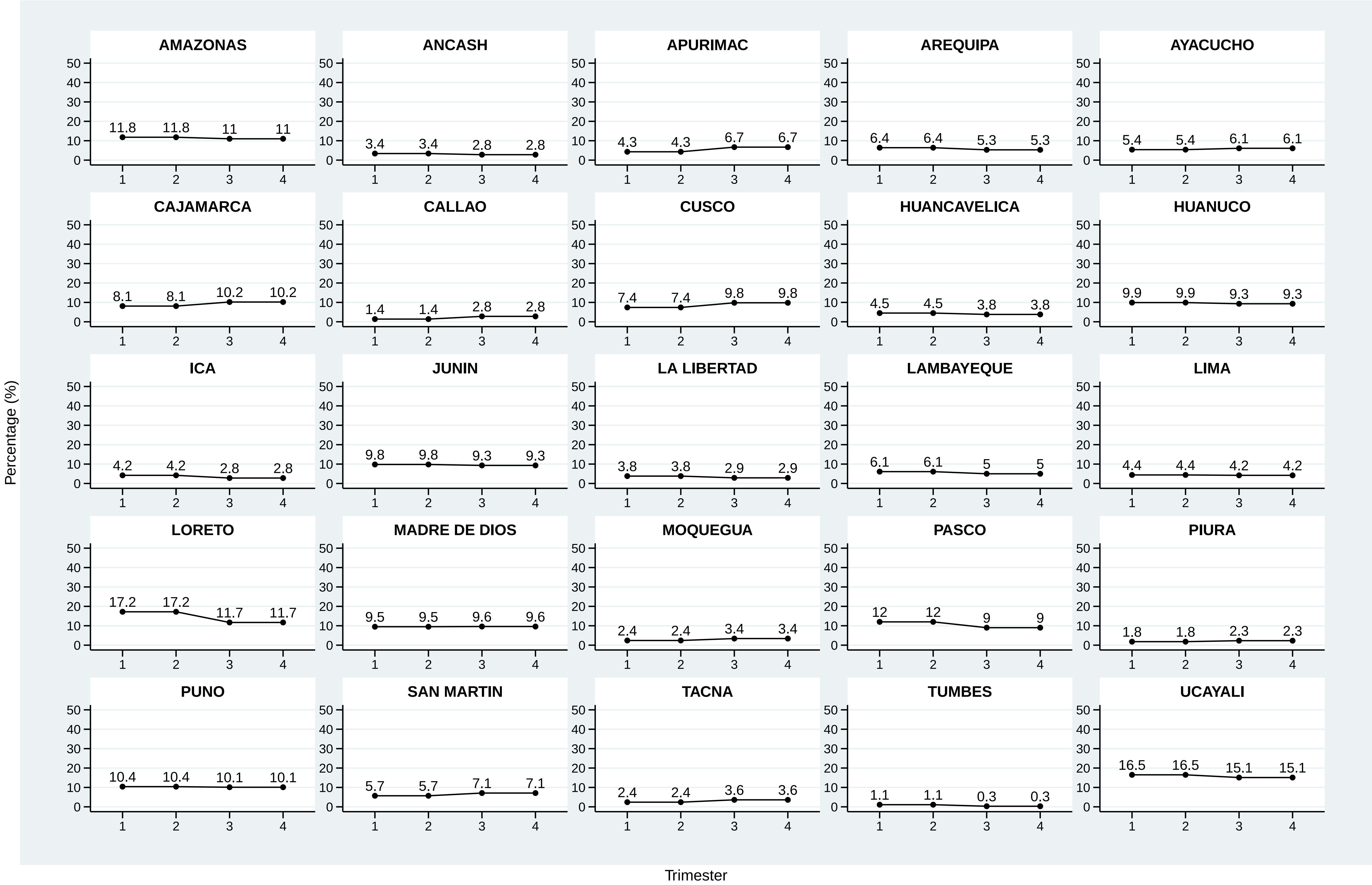

Access to improved water sources (%) by region

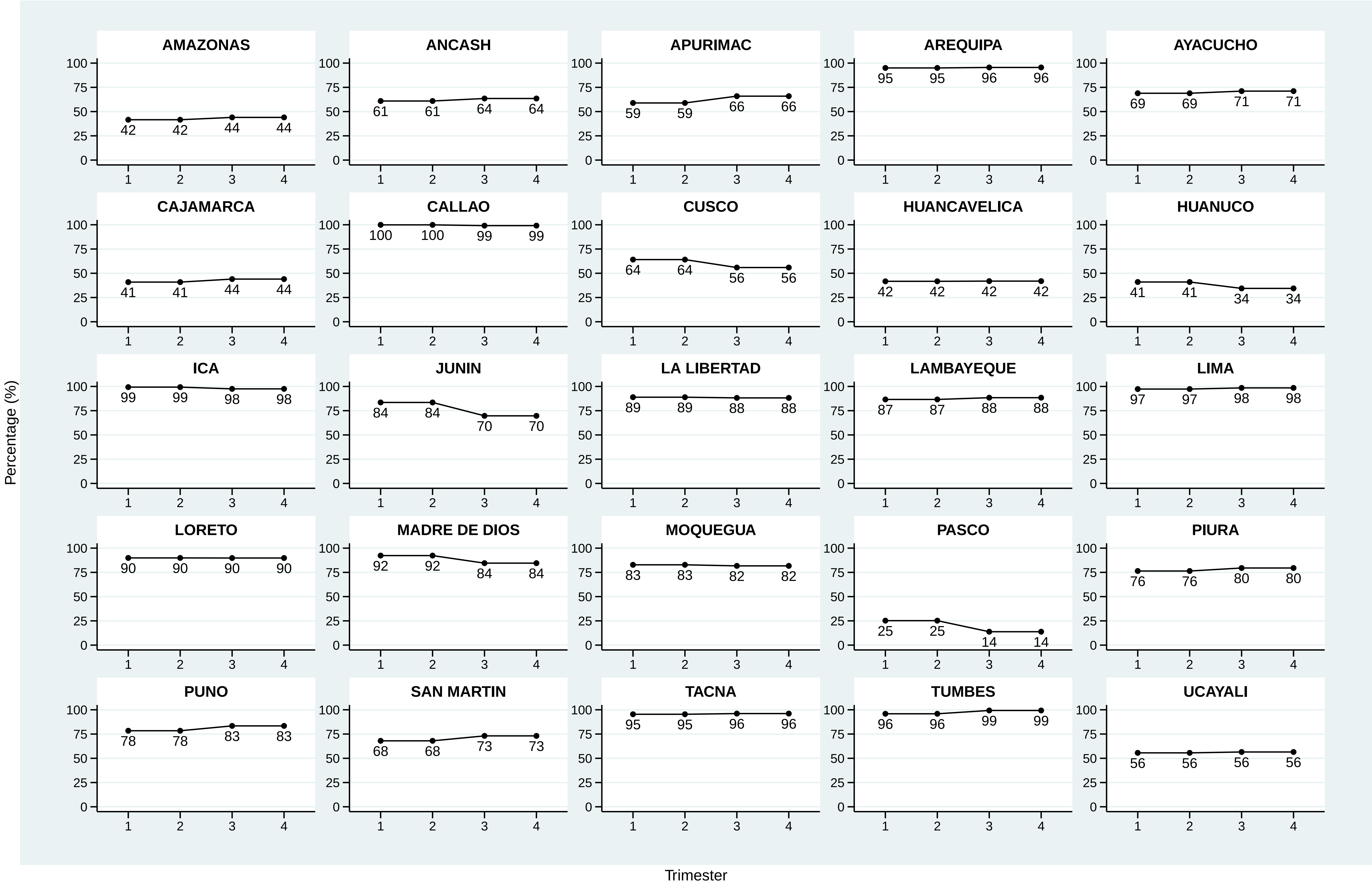

Access to improved sanitation facilities (%) by region

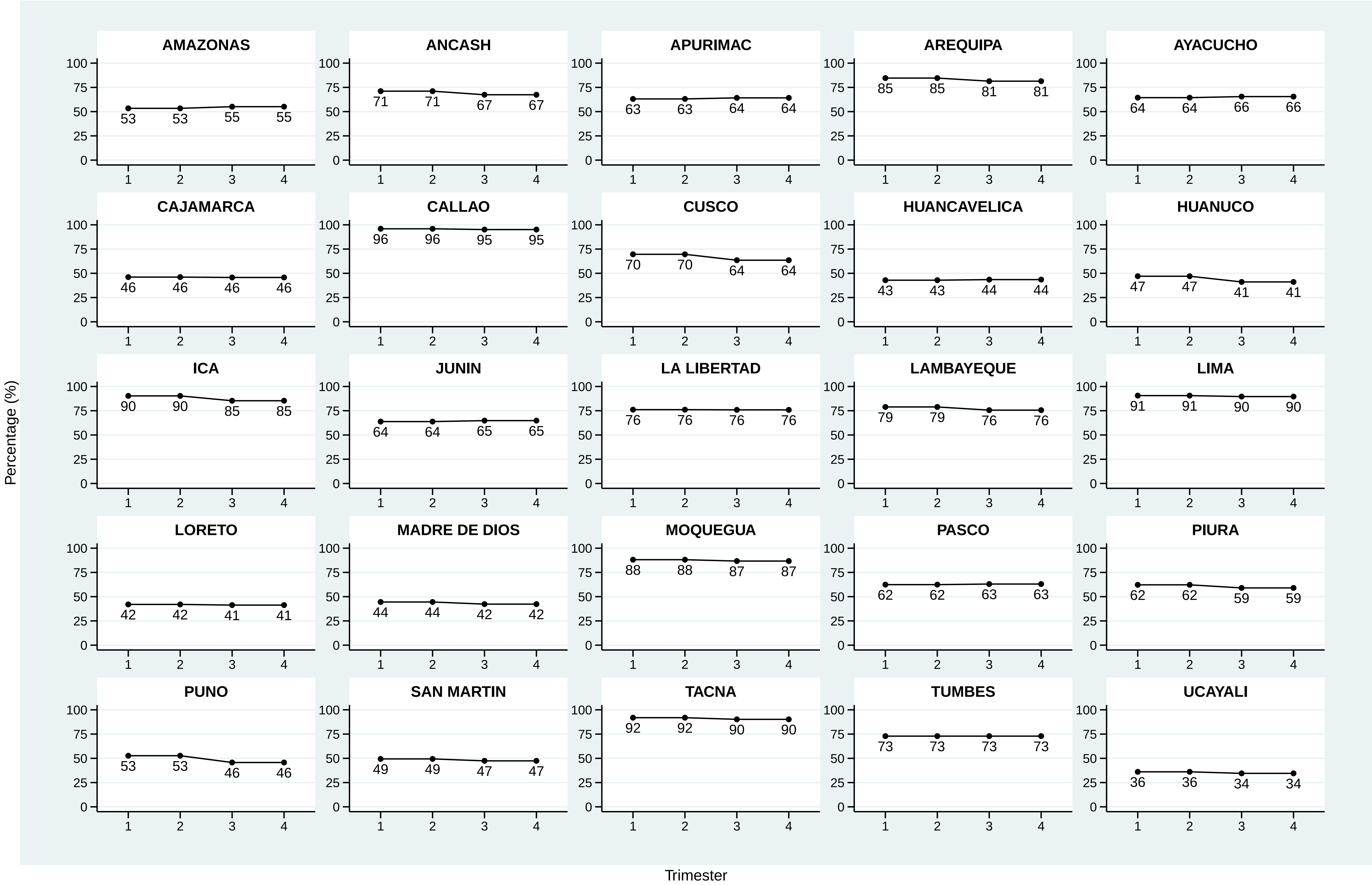

Years of schooling (median) by region

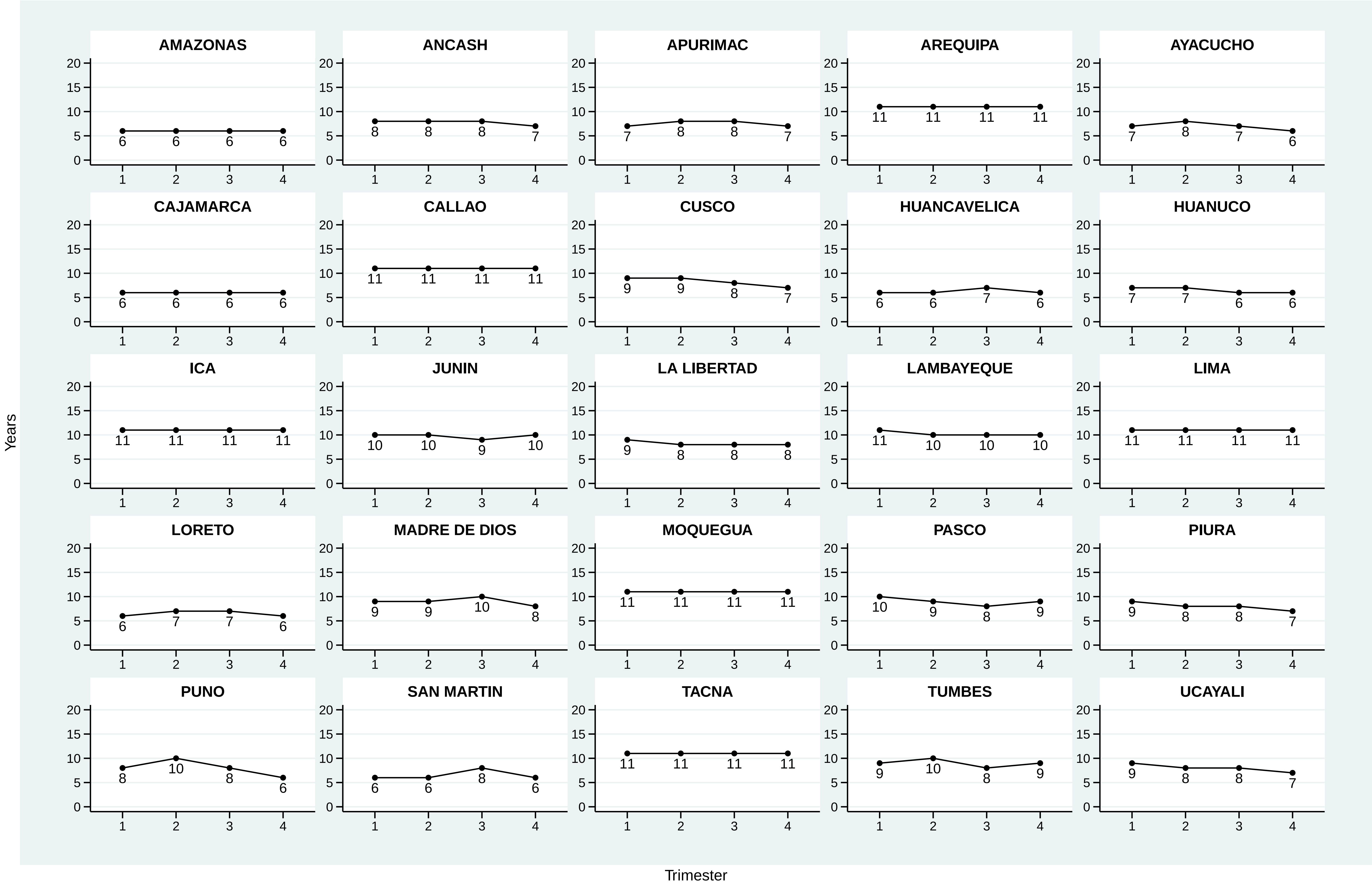

Health insurance (%) by region

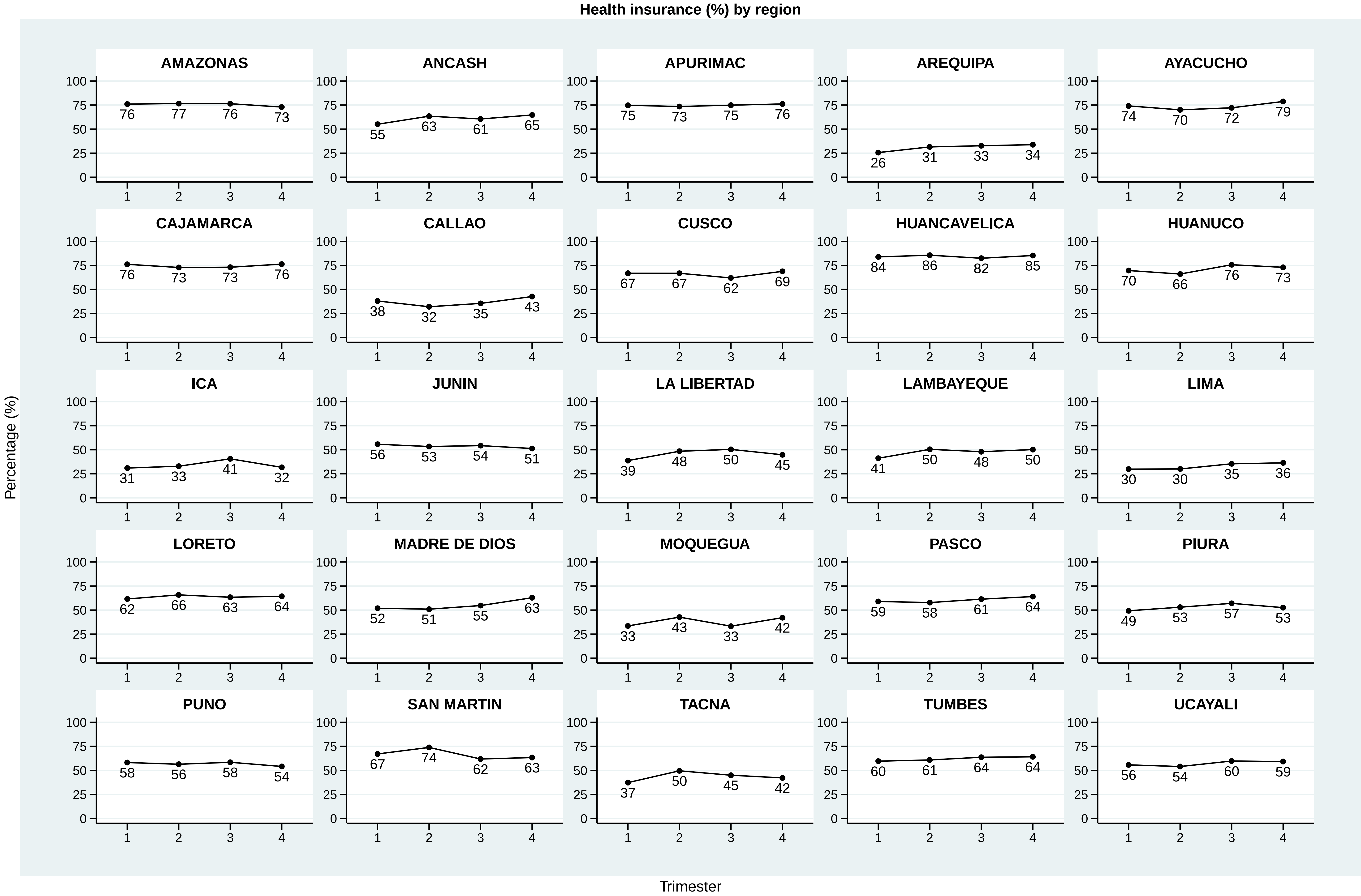

Density of human resources for health (per 10,000 population) by region

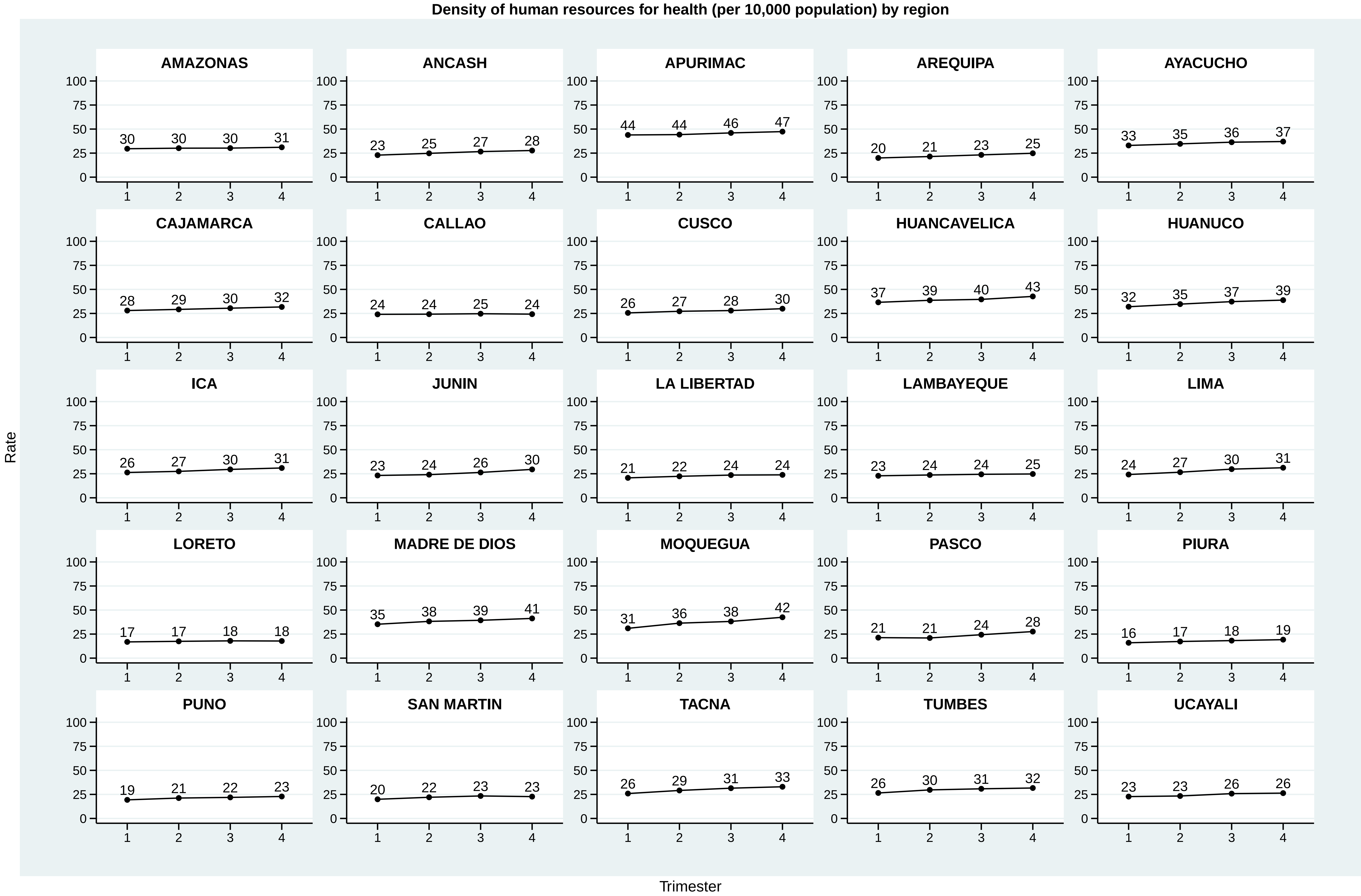

Per capita health expenditure (USD) by region

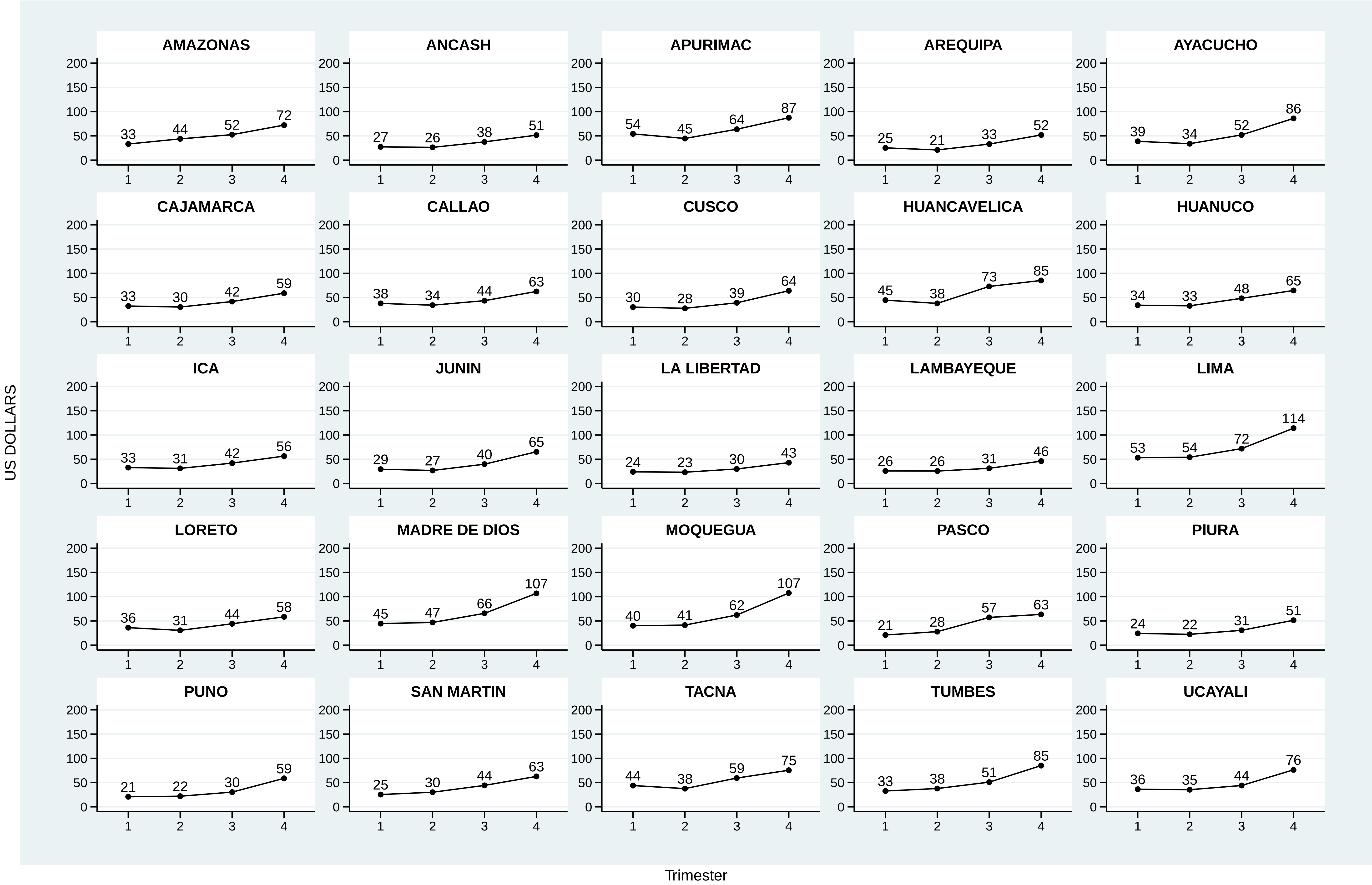

Healthcare access (per 100,000 population) by region

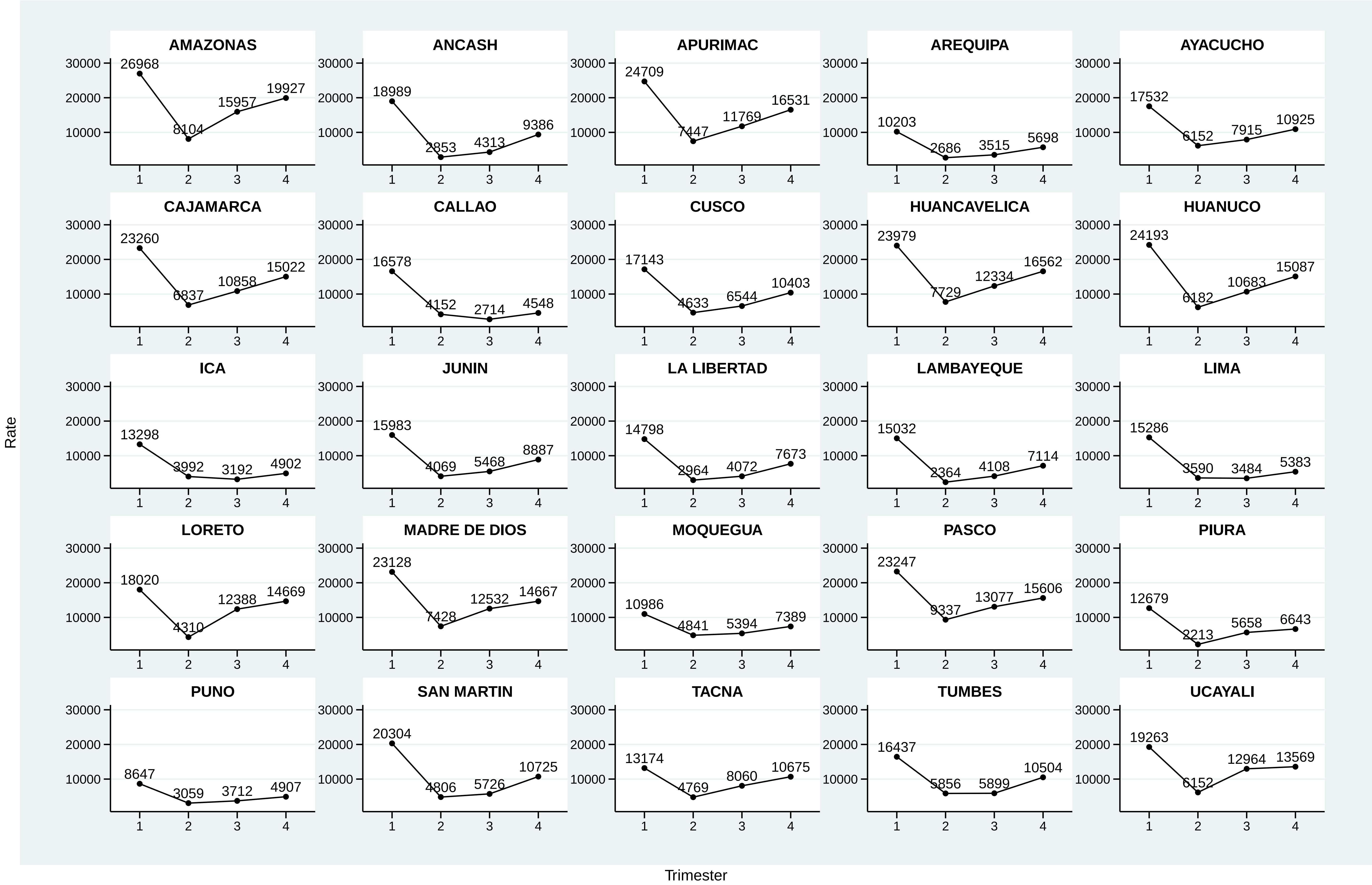

Per capita expenditure of COVID−19 pandemic health budget (USD)

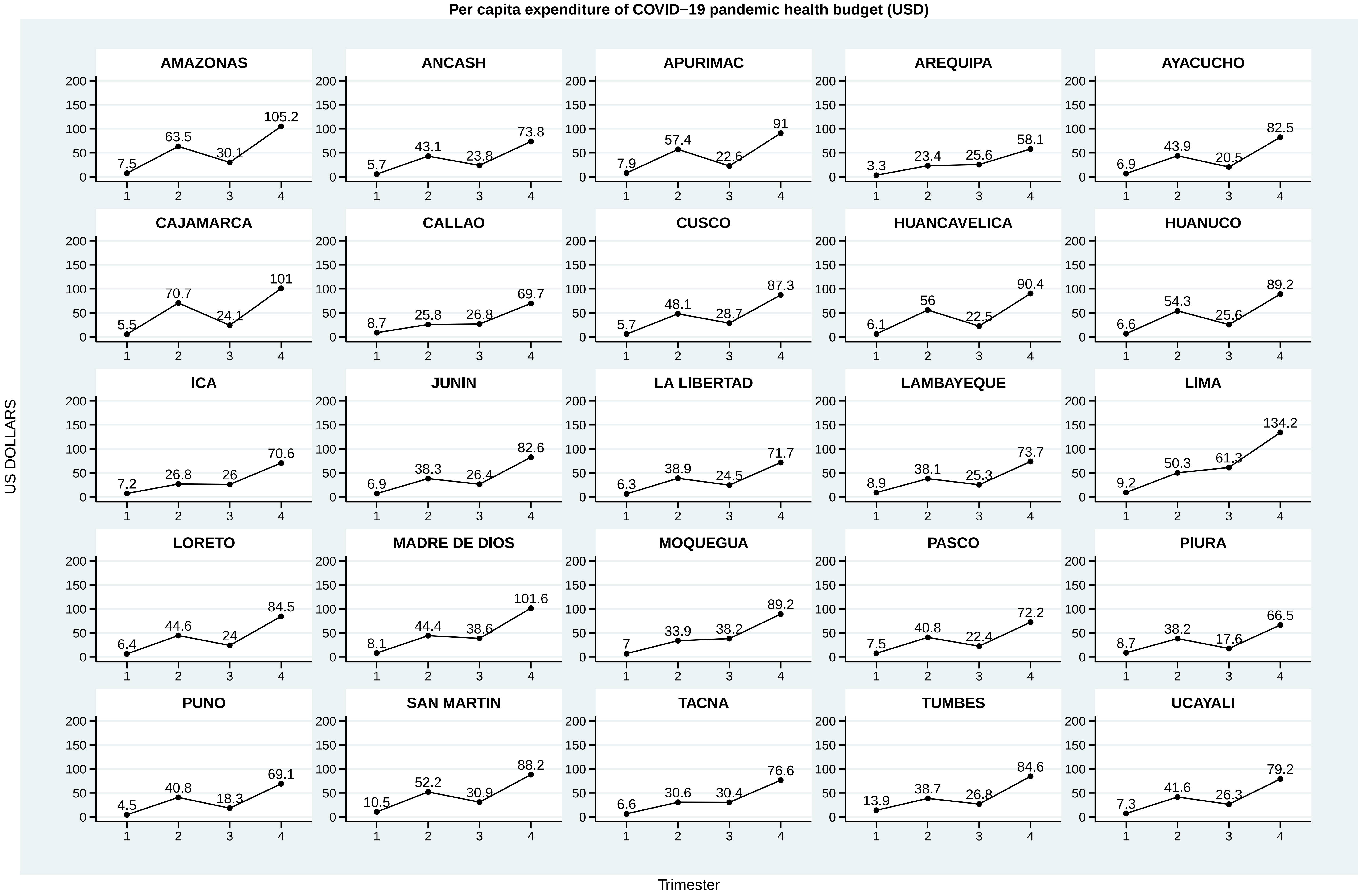

Number of COVID−19 test (per 100,000 population)

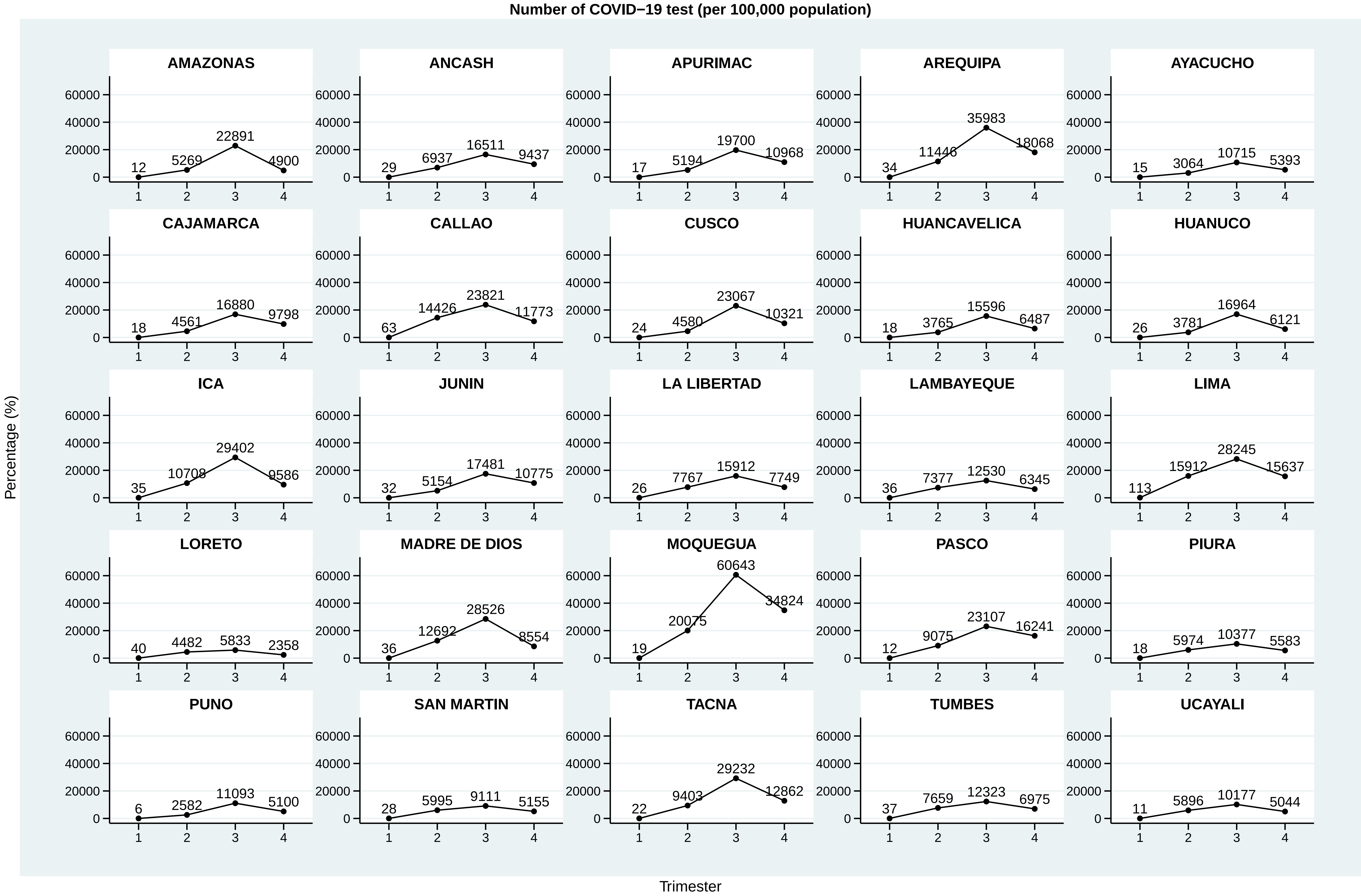

COVID−19 Incidence (per 100,000 population)

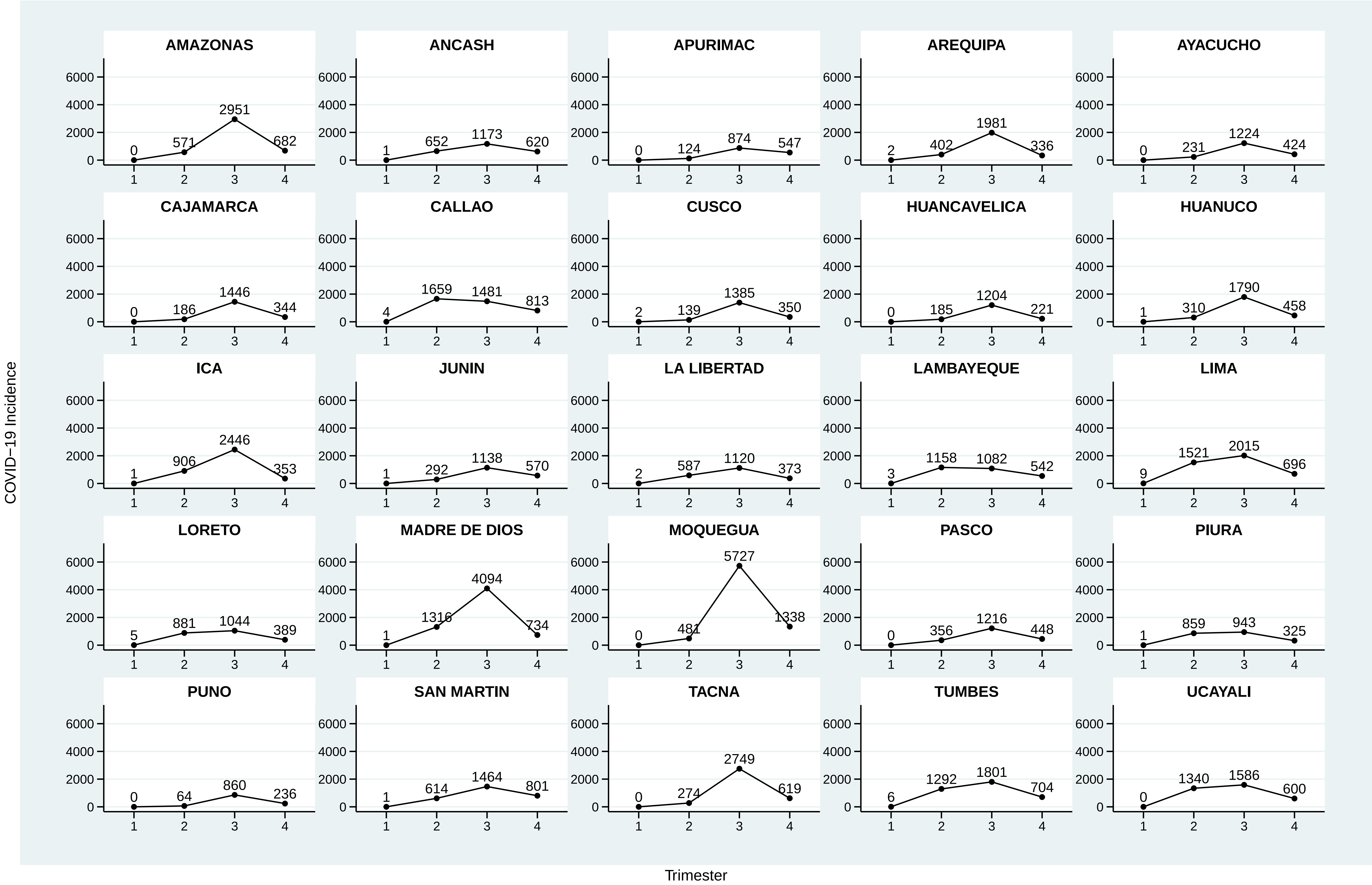

People with comorbidities (%)

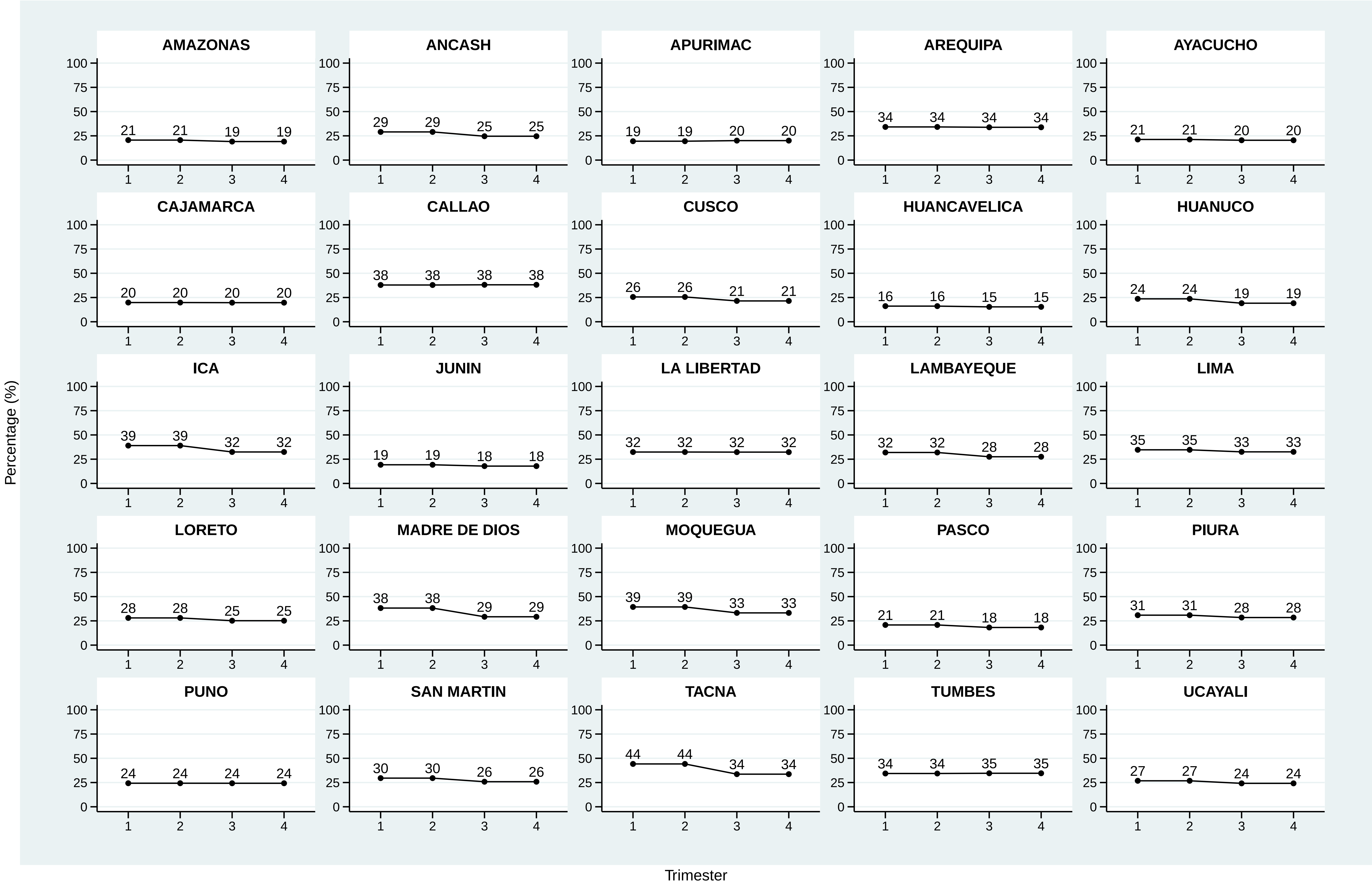

People currently smoking (%)

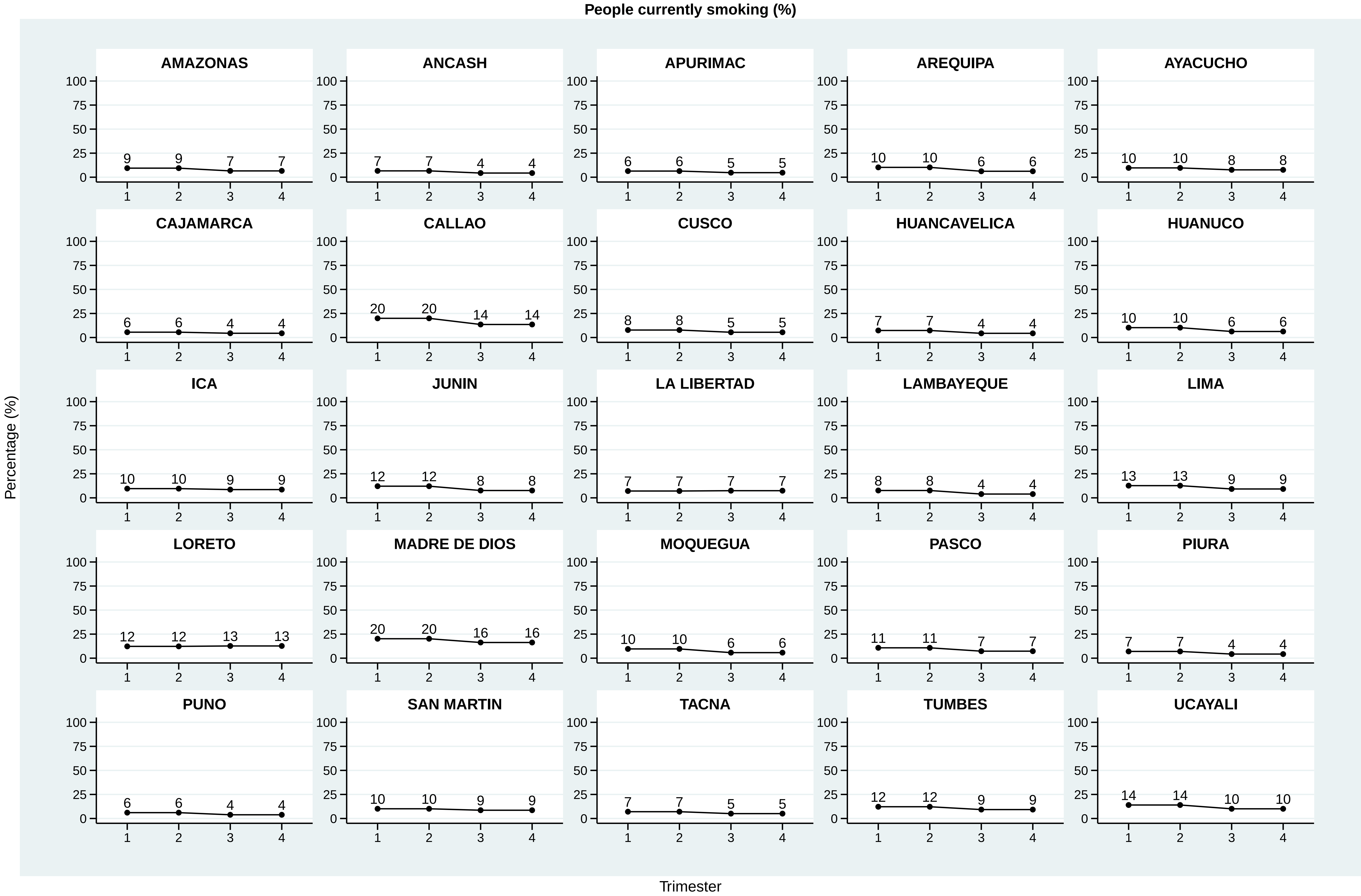

Excess death rate (per 100,000 population)

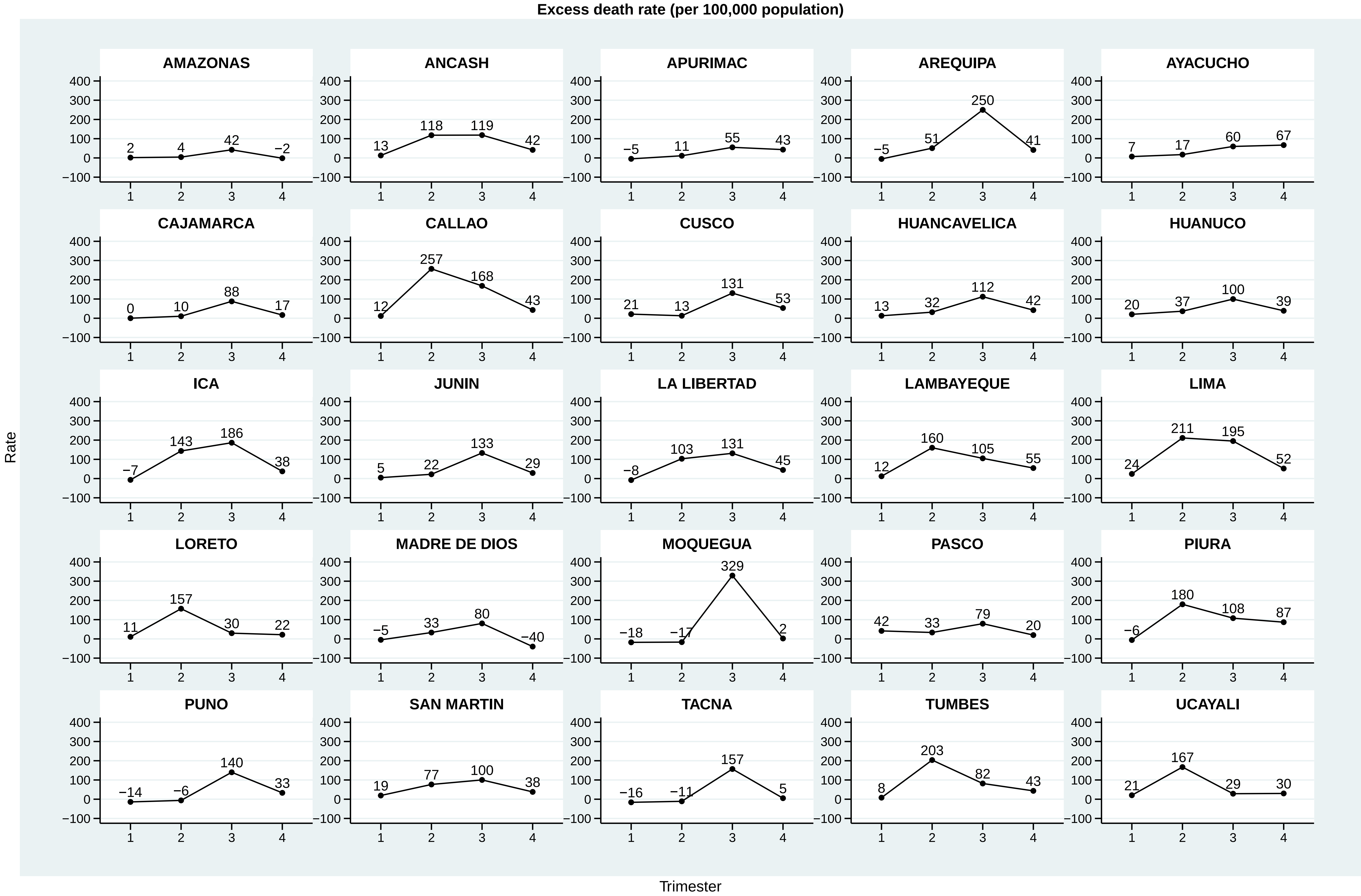

Supplementary material 3.

STROBE Statement—checklist of items that should be included in reports of observational studies

	Item No	Recommendation	Page
**Title and abstract**	1	*(a)* Indicate the study's design with a commonly used term in the title or the abstract<br>*(b)* Provide in the abstract an informative and balanced summary of what was done and what was found	1
**Introduction**			
Background/rationale	2	Explain the scientific background and rationale for the investigation being reported	2
Objectives	3	State specific objectives, including any prespecified hypotheses	3
**Methods**			
Study design	4	Present key elements of study design early in the paper	3
Setting	5	Describe the setting, locations, and relevant dates, including periods of recruitment, exposure, follow-up, and data collection	3
Participants	6	*(a)* Give the eligibility criteria, and the sources and methods of selection of participants. Describe methods of follow-up<br>*(b)* For matched studies, give matching criteria and number of exposed and unexposed	3-5
Variables	7	Clearly define all outcomes, exposures, predictors, potential confounders, and effect modifiers. Give diagnostic criteria, if applicable	3-5
Data sources/ measurement	8*	For each variable of interest, give sources of data and details of methods of assessment (measurement). Describe comparability of assessment methods if there is more than one group	5-6
Bias	9	Describe any efforts to address potential sources of bias	16
Study size	10	Explain how the study size was arrived at	6-7
Quantitative variables	11	Explain how quantitative variables were handled in the analyses. If applicable, describe which groupings were chosen and why	3-5, 7-8
Statistical methods	12	*(a)* Describe all statistical methods, including those used to control for confounding<br>*(b)* Describe any methods used to examine subgroups and interactions<br>*(c)* Explain how missing data were addressed<br>*(d)* If applicable, explain how loss to follow-up was addressed<br>*(e)* Describe any sensitivity analyses	6-8

Continued on next page

Results			Page
Participants	13*	(a) Report numbers of individuals at each stage of study—e.g., numbers potentially eligible, examined for eligibility, confirmed eligible, included in the study, completing follow-up, and analyzed<br>(b) Give reasons for non-participation at each stage	3
		(c) Consider use of a flow diagram	N/A
Descriptive data	14*	(a) Give characteristics of study participants (e.g., demographic, clinical, social) and information on exposures and potential confounders<br>(b) Indicate number of participants with missing data for each variable of interest<br>(c) Summarize follow-up time (e.g., average and total amount)	10-11
Outcome data	15*	Report numbers of outcome events or summary measures over time	11, 24
Main results	16	(a) Give unadjusted estimates and, if applicable, confounder-adjusted estimates and their precision (e.g., 95% confidence interval). Make clear which confounders were adjusted for and why they were included<br>(b) Report category boundaries when continuous variables were categorized<br>(c) If relevant, consider translating estimates of relative risk into absolute risk for a meaningful time period	10-11/Tables & Figures
Other analyses	17	Report other analyses done—e.g., analyses of subgroups and interactions, and sensitivity analyses	11
**Discussion**			
Key results	18	Summarize key results with reference to study objectives	12
Limitations	19	Discuss limitations of the study, taking into account sources of potential bias or imprecision.<br>Discuss both direction and magnitude of any potential bias	16
Interpretation	20	Give a cautious overall interpretation of results considering objectives, limitations, multiplicity of analyses, results from similar studies, and other relevant evidence	12-16
Generalizability	21	Discuss the generalizability (external validity) of the study results	12-13
**Other information**			
Funding	22	Give the source of funding and the role of the funders for the present study and, if applicable, for the original study on which the present article is based	17

*Give information separately for cases and controls in case-control studies and, if applicable, for exposed and unexposed groups in cohort and cross-sectional studies.

Note: An Explanation and Elaboration article discusses each checklist item and gives methodological background and published examples of transparent reporting. The STROBE checklist is best used in conjunction with this article (freely available on the Web sites of PLoS Medicine at http://www.plosmedicine.org/, Annals of Internal Medicine at http://www.annals.org/, and Epidemiology at http://www.epidem.com/). Information on the STROBE Initiative is available at www.strobe-statement.org.
